# Report on the Progress of Practical Medicine in the Departments of Midwifery and the Diseases of Women and Children, during the Years 1844-5

**Published:** 1845-10

**Authors:** Charles West

**Affiliations:** Member of the Royal College of Physicians; Physician to the Royal Infirmary for Children; Lecturer on Midwifery at the Middlesex Hospital; and Physician-accoucheur to the Finsbury Dispensary


### REPORT ON THE PROGRESS OF PRACTICAL MEDICINE,
### IN THE DEPARTMENTS OF
### MIDWIFERY AND THE DISEASES OF WOMEN AND CHILDREN,
*During the Years* 1844-5.

BY CHARLES WEST, M.D.,
Member of the Royal College of Physicians, Lecturer on Midwifery at the Middlesex Hospital, Physician to the Royal Infirmary for Children, and Physician-Accoucheur to the Finsbury Dispensary.

---

THE period included in this Report extends from the 1st day of January 1844, to the last of June 1845; and its plan and general character are the same as those of the last Report.

### I. ON THE PROGRESS OF MIDWIFERY.

New editions have appeared of the manuals of Dr. Ramsbotham,* and M. Chailly,† and a translation of the latter work by Dr. G. S. Bedford, of New York, has been published in America. The value of Dr. Ramsbotham's work has been increased by the addition of an essay on the diseases of the pregnant and puerperal states, and on abortion. M. Chailly's treatise has likewise undergone some enlargement; and a good alphabetical index has been added, which was a desideratum in the former edition. The American translation contains some judicious notes and additions by Dr. Bedford. The work of M. Moreau has likewise appeared in an English dress, edited by Dr. Goddard, of Philadelphia. Professor Trefurt‡ of Göttingen has published a volume of essays and observations on subjects connected with practical midwifery and the diseases of women, which shows much learning and practical knowledge. Some of the subjects of which the writer treats will be noticed in the course of the Report.

#### PREGNANCY.

*Signs of pregnancy.* Observations have been made by MM. Möller§ and Kleybolte‖ on the value of kysteine in the urine as a sign of pregnancy. The former gentleman does not attach much importance to it, since he found a very thin pellicle of it in the urine of two women who were not pregnant. In another case in which a woman was pregnant no kysteine was formed in the urine, while the person was suffering from a cold which was attended with a

* Principles and Practice of Obstetric Medicine and Surgery, 2d edition; London, 1844, 8vo.
† Traité Pratique de l'Art des Accouchemens, 2ieme ed. 8vo; Paris, 1845.
‡ Abhandlungen und Erfahrungen aus dem Gebiete der Geburtshülfe, etc. 8vo; Göttingen, 1844.
§ Casper's Wochenschr. Jan. 11-18, 1845. ‖ Ibid. April 26, 1845.

large deposit of lithic acid, but it reappeared on the urine again becoming natural. From ten observations on pregnant women M. Kleybolte arrives at conclusions favorable to the importance of kysteine as a sign of pregnancy; but he has not examined the urine of other persons, and is therefore unable to say whether it may not be formed independent of the existence of pregnancy.

A case is related by Mr. Barbieri,* in which many of the symptoms of pregnancy occurred in a patient aged 32, who had already given birth to three children. At the supposed term of utero-gestation, the breasts being then full of milk, and the abdomen large and firm, pains conceived to be those of labour, came on. The results of percussion over the abdomen were rather contradictory, but the os and cervix uteri were quite unaltered, though the vagina was moist and relaxed, and the patient stated that the liquor amnii had been discharged. The pains, however, were less regular than those of labour, and symptoms of inflammation of some important viscus were thought by M. Barbieri to be present. For these the patient was treated very energetically, but she died on the twenty-first day. The uterus was found quite healthy and unaltered, but the peritoneum was thickened and vascular, and there was very great vascularity of the duodenum, ileum, and rectum, with ulceration of the last 12 inches of the ileum.

*Disorders of pregnancy.* M. Chailly† relates the particulars of three cases of vomiting during pregnancy, which proved fatal by its severity. In the first case the patient died in the 14th week of utero-gestation; and vomiting unattended by fever had existed for three months. There was no lesion of the stomach, but "evident inflammation" of the decidua. In the second case, death took place at the same period, and obstinate vomiting had existed from the very beginning of pregnancy. Very slight lesions were found in the stomach, but there was sanguineous engorgement of the decidua and of the uterine tissue, with softening and thickening of the uterine parietes. In the third case death took place at 4½ months, the patient being then in a state of complete marasmus, from vomiting which had existed for two months.

*Abortion.* Dr. Bond‡ recommends the employment of a pair of forceps which he has invented to remove the placenta in those cases of abortion in which its retention occurs. His forceps are ten inches long, and resemble a pair of bullet forceps, much curved, and with very long blades.

[The employment of mechanical means for removing the placenta in these cases is not new; and is generally discountenanced by those who have had the largest experience, on account of the impossibility of guiding any instrument introduced into the uterus during the early months of pregnancy.]

*Rupture of the uterus.* Dr. Prael§ relates the case of a woman who became pregnant after having undergone the Cæsarean section. At the 4th month of her second pregnancy a small ulcer formed on the right side of the abdomen, and gradually increased to the size of the hand. Near the end of pregnancy, but before labour had commenced, the uterus and abdominal integuments gave way in this situation, and the fœtus with its membranes escaped into the bed. The placenta was removed by the hand, the patient recovered well, though the cicatrization of the rupture was not complete until after the lapse of ten weeks. A singular and unique case of fatal rupture of the vagina at the end of pregnancy, but before labour had commenced, is recorded by Dr. Doherty.‖ The patient in whom it occurred had a somewhat contracted pelvis, and her vagina was in an unhealthy state as the result of the severity of her previous labour. Dr. Doherty supposes that

* London and Edinb. Monthly Journal, March 1844.

† Bull. Gén. de Thérap. Oct. 30. 1844.

‡ American Journal of Med. Science, April 1844.

§ Edinb. Med. Surg. Journal, April 1845; extracted from Allg. Repert. June 1844.

‖ Dublin Hosp. Gazette, May 15, 1845.

the accident was produced by some turn of the patient in bed, by which the uterus was suddenly inclined to the opposite side of the abdomen; when the diseased vagina gave way, being unable to bear the stress thus thrown upon it. The os uteri was found after death perfectly closed, and the rent of the vagina corresponded to the right linea ileo pectinea, which, however, was not sharper or more prominent than natural.

*Extra-uterine pregnancy.* Dr. G. A. Carus* has published a paper on *interstitial* or *tubo-uterine* pregnancy, supplementary to his dissertation on the same subject which appeared in the year 1841. In this paper he relates more or less fully the particulars of fifteen cases, of which he has either found a description in medical writings, or has seen specimens in anatomical collections. He distinguishes two varieties of this form of pregnancy: 1st, *Graviditas utero-tubaria,* in which the ovum reaches only to the point where the tube begins to enter the substance of the uterus, and by its development distends only the tube—not any part of the womb; and 2d, the *Graviditas tubo-uterina, in substantia uteri, sive interstitialis,* in which the ovum is developed in that portion of the tube which is actually surrounded by the uterine substance. Of the former occurrence, he relates three cases, the remaining twelve belonging to the second class, and being specimens of true interstitial pregnancy. [The chief merit of the paper, consists in its being a very complete and clear description of all known cases of this unusual form of extra-uterine pregnancy.]

*Cases of fallopian pregnancy* are detailed by Mr. Elkington,† M. Velpeau,‡ and Dr. Lietch.§ Mr. Elkington's patient survived for more than four years, and death then took place by the accidental strangulation of a fold of the ileum between two bands of omentum which were adherent to the cyst. M. Velpeau's patient died at the end of two years, in consequence of a puncture of the cyst through the walls of the vagina and the supervention of peritonitis. Dr. Lietch's patient died with symptoms of stone at the end of five years. The ovum had originally occupied the right fallopian tube, but had escaped into the abdomen, where it had formed adhesions with the right iliacus internus muscle on one side, and on the other had become connected with and ulcerated into the bladder. In the cavity of the bladder was a calculus of the triple phosphate which had formed around a fœtal tibia. Charleton, Wheatcroft, Bacchêtti, v. Dam, Hiller, and Hemard,|| have each observed a case of *abdominal* pregnancy. In all these cases the patients died, though not always from the immediate consequences of the occurrence. In Charleton's case death took place at the 9th month, pains, like those of labour, having then come on; but it appears uncertain how far the rupture of the cyst into the vagina which was found after death was the result of efforts to turn the child which were made under a misconception of the nature of the case. Life was prolonged for three years in Wheatcroft's case, the patient eventually dying from exhaustion. Fœtal bones had been passed per rectum, but it is remarkable that though the sac communicated very freely with the uterus there was at no time any discharge from the vagina. Dr. Bacchetti's patient had labour-pains at the end of the regular term of pregnancy which subsided in the course of a fortnight, she afterwards gave birth to two living children, and it was not till ten months after the birth of the second, that the abdominal tumour grew soft, and that fever and diarrhea came on, which destroyed life five years and three months after the occurrence of the extra-uterine conception. v. Dam's case is not very clearly described; but it appears to have been an instance of natural pregnancy, in which rupture of the uterus took place during labour,

* Neue Zeitschrift f. Geburtskunde, Bd. xv, p. 161. † Provincial Med. Journal, Jan. 8, 1845.
‡ Gaz. des Hôpitaux, Mai 6, 1845. § Lond. and Edinb. Monthly Journal, Feb. 1845, p. 106.
|| Med. Gazette, Feb. 16, 1844; Lancet, Feb. 24, 1844; Gaz. des Hôpitaux, Oct. 24, 1844; Oppenheim's Zeitschr. März 1845, p. 361; Med. Zeitung, April 16, 1845; Lancet, Oct. 12, 1844.

and the fœtus escaped into the abdominal cavity. At the end of 21 months severe pain again came on in the abdomen, which having lasted for 8 months, v. Dam performed gastrotomy and removed the fœtus. In 17 months the patient again became pregnant, and when in the last month of utero-gestation pain came on in the situation of the cicatrix, a sac-like prominence formed at the opening in the abdominal muscles, and the woman died in a state of collapse; the fœtus having passed through a laceration in the cyst which contained it, and which was situated behind the uterus, and formed to a great extent the posterior wall of the organ. In Dr. Hiller's patient, the fœtus lay between the uterus and rectum, and was partly driven by the violent expulsive efforts through a rent in the vagina, which is said to have taken place without any manual interference. The patient died undelivered. Mr. Hemard's patient died exhausted at the end of three years, having passed some fœtal bones by the urethra, and communications were found to exist between the cyst containing the fœtus and the bladder.

### NATURAL LABOUR.

*Management of labour.* Dr. W. Smith,* in an ingenious but rather prolix paper, dwells on the importance of altering the position of a woman during labour, according to the degree of inclination of her pelvis. He attaches, though chiefly on theoretical grounds, very considerable importance to obliquity of the uterus as a cause of protracted labour, and conceives that by changing the woman's position the axis of the uterus and that of the pelvis may be made to coincide. [Two fallacies lie at the bottom of Dr. Smith's argument: first, the assumption that the relation of the pelvis to the lower part of the vertebral column may be materially altered by the woman's position,—a thing clearly impossible owing to the very slight mobility of the pelvis backwards or forwards on the spine; and second, the misapprehension of the relation between the axis of the uterus and that of the pelvis, which will always be found to correspond, except in some cases of great deformity of the pelvis, or where, from a relaxed state of the abdominal integuments, pendulous belly exists.†

Dr. Breen‡ advocates the late Dr. Hamilton's practice of artificially dilating the os uteri, and thus abridging the duration of labour in cases where the first stage is tedious. He does not adduce much new evidence in favour of the practice, but the mere opinion of one who has had large experience is entitled to some weight.

Dr. Beatty§ and Dr. Hardy|| have published the results of some researches upon the influence of the ergot of rye on the mother and the child. Dr. Beatty thinks that the powers of this drug have generally been either over or underestimated. He believes, however, that it exerts a poisonous influence on the fœtus, which becomes evident if the child be not born within a certain period, which he fixes at three hours after its administration. He conceives that it destroys the child not merely by exciting violent uterine action, but by its directly poisonous properties, since many children who were not still-born after its employment had peculiar convulsive affections which continued in some instances for several days. These convulsions too were very dissimilar from those which occur in any form of asphyxia neonatorum, while their resemblance to those which have attended ergotism in the adult further support this opinion. Dr. Hardy entertains similar views to those of Dr. Beatty with reference to the influence of the ergot on the fœtus, since the child was still-

* Edinburgh Medical and Surgical Journal, Oct. 1844.

† On this subject the reader may consult with advantage Boer's observations, De obliquitate uteri, in his Septem Libri de Obstetricia Naturali, lib. ii, cap. i; and Naegele's at § xiii, of his book, Das weibliche Becken—where are two remarkable cases of extreme deviation from the natural inclination of the pelvis, without any disturbance of the perfectly natural course of labour.

‡ Lancet, May 11, 1844. § Dublin Journ. of Med. Science, May 1844. || Ibid. May 1845.

born in 33 out of 47 cases in which the ergot was given, and in 15 of these 33 no instrumental interference whatever was resorted to, to which its death could be attributed. Dr. Hardy found that the action of the drug begins to be perceptible in 15 minutes on the average, that in 19 out of 47 cases it caused a diminution in the frequency of the mother's pulse which often continued for several days. The pulse of the fœtus was still more frequently diminished in frequency, and in those cases where the fœtus died it likewise became irregular, then intermittent, and finally ceased.

*Signs of delivery.* Dr. Cormack* has examined into the value of the dark abdominal line noticed by Dr. Montgomery† as occurring in some instances after parturition, and to which Mr. Turner‡ attached great importance as a sign of recent delivery. Dr. Cormack has almost always observed it after delivery at the full time; but he has likewise seen it during menstruation and pregnancy and after abortion. He has also noticed it in women who were in none of the above conditions, and who had no affection of the uterine system, as also in a few instances in men. He hence attaches to it but little diagnostic value; a conclusion in which Dr. Montgomery,§ in a communication on the same subject, coincides. The latter mentions a dark areola round the umbilicus, which he has occasionally seen after mature delivery, but not at other times, though he does not imagine it to be limited to that period.

*Plurality of children.* A case of triplets is related by Mr. Arman,|| in which the children, two girls and a boy, were all living. Three instances are likewise recorded in which women had four children at a birth. In the first case¶ the children were all boys and all born alive, but died on the fifth day. In the second,** the mother had previously given birth to triplets—two boys and a girl. In the succeeding labour she had three boys and one girl. One boy died on the following day; but the other children survived. In the third case†† two of the children were male, and two were female; they were all born alive, and were thriving thirteen days after birth.

Dr. Pfau‡‡ relates a case of twin labour in which there was complete suspension of uterine action for seven days, between the birth of the first and second child. A similar case, but in which the interval was thirty-two days, is recorded by Mr. Irvin;§§ and a third is related by Dr. Wildberg,|||| in which it extended to two months. In this last case no milk was secreted till after the birth of the second child, when it at once became very abundant.

### PRETERNATURAL LABOUR.

*From causes depending on the mother. Abnormal states of the uterus.* Dr. Pellegrini¶¶ relates a case in which labour was protracted by a great enlargement of the anterior lip of the uterus, which shrank to a third of its former size directly after the extraction of the child, and totally disappeared by the fourth day after delivery. Dr. Pellegrini supposes it to have been a *varix of the os uteri.* A case is related by Dr. Bedford,*** in which it was necessary to incise the os uteri during labour, in consequence of its edges having become agglutinated by inflammation, the result of attempts on the part of the patient to induce abortion by mechanical means. Labour had continued ineffectually for 29 hours, but within ten minutes after the os was incised the child was

* Lond. and Edinb. Monthly Journ. Feb. 1844.
† Signs and Symptoms of Pregnancy, pp. 304-7.
‡ London and Edinburgh Monthly Journal, Aug. 1842.
§ Dublin Journal, May 1844.
|| Lond. and Edinb. Monthly Journ. Nov. 1844.
¶ Pfau, Oest. Med. Wochenschr. Feb. 10, 1844.
** Anonymous writer, in Schmidt's Jahrb. Bd. xliv, No. xii, Heft 3.
†† Black, Northern Journal of Medicine, March 1845, p. 265.
‡‡ Oesterr. Med. Wochenschr. April 20, 1844.
§§ Medical Times, Dec. 28, 1844.
|||| Oesterr. Med. Wochenschr. April 5, 1845.
¶¶ Arch. Gén. de Méd. Aug. 1844.
*** New York Journal of Medicine, March 1844.

born, and the patient did perfectly well. Dr. Davis* and Mr. Reardon† have each met with a case in which the lower segment of the uterus was separated in labour; the os uteri having been perfectly rigid and undilatable. In both instances the patient did well.

Two cases of extreme *obliquity of the uterus* are detailed by Dr. Pellegrini and Dr. Bresciani di Borsa.‡ In the former case the abdomen was so pendulous that the uterus rested on the patient's thighs. Delivery was effected by turning, but the woman died of metroperitonitis on the fourth day. In Dr. di Borsa's patient the obliquity of the uterus was lateral, the womb hanging like a retort over the right ileum. This malposition was apparently owing to great deformity of the pelvis, which rendered the Cæsarean section necessary from which the patient recovered. An instance of labour, complicated with *prolapse of the uterus,* very similar to Dr. Perfetti's case recorded in the last Report, is detailed by Dr. Darbey.§

Dr. de Billi|| relates the history of a patient whose uterus became *retroverted* at an early period in her second pregnancy, and continued so, attended as utero-gestation advanced with very urgent symptoms, until the middle of the eighth month. A discharge of fluid then took place, and three days afterwards labour-pains came on. Externally the uterus seemed of natural size and form, but a large round tumour was felt between the uterus and rectum, and the os uteri was very high up, tilted five fingers' breadth above the pubes, quite beyond the reach of the hand. Notwithstanding this misplacement the patient had always voided her urine, though with great difficulty. Dr. de Billi reduced the uterus by pressure on its fundus, exerted through the vagina, while counter-pressure was made externally. The child, which presented by the breech, was stillborn, but the mother recovered well. [This case fully substantiates the accuracy of Dr. Merriman's observations in his Dissertation on Retroversion of the Womb, and adds another to the very small number of cases in which the womb has continued retroverted up to the commencement of labour.]

*Inversion of the uterus.* The first part of a very valuable essay on this subject has appeared from the pen of Mr. Crosse.¶ The simple detail of facts has been his object in this part; but he promises in the second to consider the causes, diagnosis, and treatment of the affection. He proposes the terms depression, introversion, perversion, and total inversion to designate the different degrees of this accident, and describes minutely the mechanism of its occurrence, during parturition. Its symptoms and immediate consequences are stated less fully, but will be examined in the second part of the essay. Cases of spontaneous inversion of the uterus during labour are recorded by Messrs. Barker, Crosse, Clarkson, and Edwards.** In Mr. Barker's case the accident occurred about twenty minutes after the expulsion of the placenta; the hemorrhage was profuse, but the uterus was reduced in the course of half an hour. The inversion was produced in Mr. Clarkson's patient by the same pain as expelled the child, and detached the placenta. There was great depression of the system, but not very severe hemorrhage: and the uterus was easily reduced. In Dr. Edwards's patient the inversion occurred after the expulsion of the child, the placenta being still partially adherent, and the hemorrhage very profuse. The uterus was reduced after the removal of the placenta, considerable internal hemorrhage subsequently occurred, but the patient recovered. Mr. Crosse's case is interesting from the same accident having occurred once before to the patient. On both occasions it was connected with morbid adhe-

* Dublin Med. Press, Jan. 15, 1845.
† Ibid. March 19, 1845.
‡ Archives Gén. de Médecine, Fevr. 1845.
§ Dublin Medical Press, Nov. 6, 1844.
|| Annali Univ. di Med. Febb. 1845, p. 312.
¶ An Essay on Inversio Uteri; in Trans. of the Prov. Med. and Surg. Association, vol. xiii, p. 285.
** Medical Gazette, April 5-12, 1844; Prov. Med. and Surg. Journal, June 12, 1844; Lancet, Dec. 28, 1844: Ibid. April 5, 1845.

sion of the placenta, which body was removed by the hand. On the second occasion of its occurrence, attempts at reposition instituted after the lapse of an hour were quite unsuccessful. In the cases related by Mr. Square, Dr. Esselman, Dr. Gazzan, and Dr. M'Clintock,* it is either expressly stated, or may be fairly assumed that traction had been made by the funis to remove the placenta. The first of these cases shows the importance of not omitting to make a vaginal examination whenever considerable hemorrhage occurs, and continues after labour; since from that omission the inversion was not detected till ten days after delivery, at which time it was found to be irreducible. Dr. Gazzan succeeded in reducing the uterus on the ninth day, having previously brought the patient under the influence of antimony. In Dr. Esselman's case, and in the two related by Dr. M'Clintock, unsuccessful attempts to reduce the uterus had been made before the patients came under the care of those gentlemen, and the disease had passed into a chronic condition. The cases recorded by the four last-mentioned writers are important, inasmuch as they show the possibility of inversio uteri, not occurring, or at least not giving rise to well-marked symptoms until some hours or even days after delivery.

Dr. Oldham† relates six cases of labour complicated with *polypus uteri,* five of which have not been published before, none of them, however, occurred under his own observation. He notices the difficulty of ascertaining the presence of polypi during pregnancy, and the fact that they by no means constantly derange the process of labour. The most dangerous symptom to which under these circumstances they may give rise, is serious hemorrhage. He recommends that unless such hemorrhage exist, no attempt be made to remove the polypus immediately after delivery. The cases he relates illustrate the differences with respect to this point of practice which different symptoms may render necessary. M. Aubinais‡ relates a case in which the introduction of the hand to remove an adherent placenta gave rise to the discovery of a polypus of the size of an egg, to which the placenta was attached, and from which it could not be separated. The pedicle of the polypus was easily twisted off, and no hemorrhage followed its removal. No peculiar symptom indicative of its presence had existed during pregnancy.

A case of labour obstructed by a *longitudinal septum dividing the vagina* into two cavities, only one of which communicated directly with the os uteri, is related by M. Lesaing.§ The patient was safely delivered after the septum had been divided. Mr. Headland|| has observed a case in which the birth of the child was obstructed by the presence of a large malignant ovarian cyst in the recto-vaginal pouch. The cyst burst under the pressure of the child's head, and delivery was effected by the forceps, but death took place in twenty-four hours. An instance of *osteosteatomatous tumour of the pelvis,* causing an impediment to the passage of the child, and requiring the performance of craniotomy is related by Dr. Pellegrini.¶ [Of all the causes of obstructed labour, none are so rare as the presence of osteosteatomatous tumours in the pelvis, of which two other observations only are on record; in Puchelt, de Tumoribus, etc. p. 48.]

*Rupture of the uterus, and laceration of the vagina.* References are contained in the note to several fatal cases of rupture of the uterus or vagina, all of which occurred spontaneously independent of any manual interference, or of the existence of disproportion between the mother and child.** In five of

* Provincial Medical and Surgical Journal, July 24, 1844; American Journal of Medical Science, Jan. 1844; Ibid. April 1844; Dublin Journal, May 1845.

† Guy's Hospital Reports, New Series, vol. ii.

‡ Gaz. Médicale, Sept. 7, 1844.

|| Lancet, June 22, 1844.

¶ Archives Gén. de Méd., Aug. 1844.

** Rendell, Med. Times, May 4, 1844; Arnold, Prov. Med. and Surg. Journal, July 24, 1844; Wright, Boston Med. Journal, June 1844; Elkington, Prov. Med. and Surg. Journal, Sept. 11, 1844; Griscom, New York Journal of Medicine, May 1844; Adler, Neue Zeitschrift f. Geburtsk. Bd. xvii, Heft 1; Bond, American Journal of Med. Science, Jan. 1845; Pavetti, Gaz. des Hôp., 14 Juin 1845.

these cases, (those related by Messrs. Rendell, Arnold, Bond, and Pavetti,) softening of the uterus appears to have preceded the occurrence of the rupture; but in the other four it is difficult to assign any cause as having predisposed to the accident. In Dr. Wagstaff's case* two exostoses on either side of the symphysis pubis appear to have caused the accident; in the cases recorded by M Laborie, and Professor Trefurt,† the uterus gave way during the operation of turning, and in the instances related by Dr. Fahnestock and Dr. Feldmann,‡ the child was hydrocephalic. In these cases likewise the patient died. Drs. Brühl, Majer, C. H. Kühn, Graus, and Morgan,§ have detailed cases of recovery after alleged rupture of the uterus. Dr. Brühl's case is not very clearly reported; but it appears that the treatment adopted by him as well as by Dr. Majer was decidedly antiphlogistic. The injury in Dr. Majer's case consisted in laceration of nearly half of the vagina from the cervix uteri; but the rent healed speedily, and the patient left her bed in four weeks. In Dr. Kühn's case some of the symptoms of ruptured uterus having occurred, he nevertheless administered the ergot of rye, and repeated it at intervals during eight hours. At the end of that time, the child passed into the abdominal cavity, on which he performed gastrotomy, and having removed the child, the woman recovered without any very grave symptom. M. Graus's case is a still more extraordinary instance of recovery after the most unwarrantable interference, such as injecting fluid into the abdominal cavity through the laceration in order to favour the escape of pus. It is by no means clear that Dr. Morgan's was a case of rupture of the uterus; no sign of rupture occurred during labour, and when seen by Dr. Morgan thirty-six hours after delivery, the patient was suffering from constipation of ten days' standing, and indications of abdominal inflammation. Blistering the abdomen, the use of calomel and purgatives were succeeded by amendment, though the uterus continued large, hard, and painful. Ten days after delivery, violent hemorrhage occurred per vaginam, and after the lapse of another seven days, the fæces began to pass by the vagina, and continued to do so for thirty days. The patient gradually recovered.

Trefurt|| makes some observations on *sanguineous tumours of the labia* occurring during labour, and relates the particulars of a case which terminated fatally in the course of a few minutes from rupture of the labium, and uncontrollable hemorrhage. In this case the veins of the thigh and of the labia appear not to have been varicose, and no reason could be assigned for the accident. The same writer has collected all recorded instances, fourteen in number of *disjunction of the symphysis of the pubis*¶ during labour, and has added the particulars of an instance of the kind which occurred in his own practice. The patient was affected with mollities ossium, and consequent deformity of the pelvis; the forceps were applied to accomplish delivery; and though no great force was used, yet in consequence of the diseased state of the parts the symphysis pubis was separated, the ligamentum arcuatum torn through, and the connexions between the sacrum and the ossa inominata were loosened.

*Preternatural labour, from causes depending on the child or its appendages.* Dr. Simpson** in an extremely interesting paper, has investigated the influence of the *sex of the child* on labour. After confirming the late Dr. Clarke's statements with reference to the larger size of the head of the male fœtus, he endeavours to show that the difficulties of parturition, and the dangers both to mother and

* New York Journ. May 1844. † Gaz. des Hôp. July 20, 1844; Abhandlungen, etc. p. 301.

‡ New York Journal, May 1844; Med. Zeitung, No. 10, 1844.

§ Casper's Wochenschrift, 18 Mai, 1844; Schmidt's Jahrb. Bd. xliii. No. viii, p. 47; Oesterr. Med. Wochenschr. Oct. 12, 1844; Dr. Ranking's Retrospect, p. 168; and American Journal of Medical Science, April 1845, p. 521.

|| Op. cit. p. 314. ¶ Ibid. p. 180. ** Edinb. Medical and Surgical Journal, Oct. 1844.

child are greater in male than in female births, and that no reason exists for this difference, other than that furnished by the larger size of the male head. His ingenious tables cannot be given in this Report, but the conclusion at which he arrives is that there is an excess of male children, to the amount of 20 per cent. among stillbirths, owing to the larger size of the male cranium; and that from the same cause among women dying during or after labour, the proportion who have given birth to male children is to those who have given birth to females as 168 to 100.

Dr. Lecluyse* relates the case of a woman in whose labours the *arm* of the child *presented* thrice in succession, an occurrence that he attributes to the peculiar form of the uterus which had become remarkably developed in its transverse diameter. Mention may be made of the publication of a second edition of Dr. Douglas's pamphlet on the process of Spontaneous Evolution; to six cases† of which references are given in the note. In all of these cases but the last, the children were stillborn, and in that the child survived but a very short time.

Trefurt‡ is very earnest in his recommendation of turning by one foot in all cases, and advises that no attempt should be made to seize the second except where it is found impossible to turn without so doing. He examines the history of the practice, and states the different arguments against the operation very fully and candidly, as well as those in its favour. As far as the preservation of the life of the child is concerned, his results are rather more favorable than usual, since the proportion of living children that he obtained was 43 per cent. Dr. Simpson,§ following the advice of some writers, prefers turning by the knee whenever it is practicable to seeking for the feet. He recommends that on all occasions the foot or knee of the side opposite the presenting part should be seized. He conceives that by so doing the rotation of the child on its long axis is greatly facilitated, a manœuvre by which the removal of the presenting arm from the os uteri is effected.

Dr. Walter‖ relates the history of a patient whose delivery was impeded, she being pregnant with twins, by the head of the second child which presented naturally, resting against the chest of the first child which presented by the breech, and thus preventing its expulsion. He eventually succeeded after much difficulty in raising the head of the second child, sufficiently to allow the other to be expelled. The first child was stillborn, but the second was born alive.

M. Hirz¶ has discussed the influence of a *twisted* or preternaturally *short umbilical cord* in delaying the passage of the head through the pelvis, and impeding delivery. He attaches great importance to it; and not only appeals to the authority of von d'Outrepont in support of his opinions, but relates three cases which came under his own observation, and which seem to bear out his views. He recommends that in these cases the forceps should be applied, and the cord divided as soon as it, or the neck of the child around which it is twisted, comes within reach.

M. Danyau** asserts that blood extravasated on the fœtal surface of the placenta sometimes becomes an organized tumour, which increases in size, interferes with the nutrition of the fœtus, and occasionally presents a mechanical obstacle to the removal of the placenta. He relates two cases illustrative of these views, of which the following was the most striking. The fœtal surface of the placenta presented near its circumference an oval tumour, subjacent to

* Annales de la Soc. de Méd. d'Anvers, Fevrier 1845.

† Danyau, Gaz. des Hôp. 18 Juin 1844; Edwards, Lancet, May 25, 1844; Wardleworth, Ibid. Aug. 3, 1844; Susewind, Casper's Wochenschrift, June 8, 1844; Casp, Med. Zeitung, May 8, 1845; Gamberini, Gaz. des Hôpitaux, Juin 7, 1845.

‡ Op. cit. p. 1-96.

§ London and Edinburgh Monthly Journal, Feb. 1845.

‖ Neue Zeitschr. f. Geburtsk. Bd. xvi, Heft 2, p. 171.

¶ Gaz. Médicale, Mai 10-17, 1845.

** Journal de Chirurgie, Janv. 1844.

the amnion and chorion 4⅓ inches long, by 3 inches broad. This tumour was slightly lobular, its lobules being closely adherent to each other; some of a dirty white, others of a pale rose tint, and of a very various texture. It was traversed by large branches of the umbilical vessels, some of which entered it, but many of these vessels were blocked up with coagula. Another smaller tumour was near it, and the placenta was so much enlarged by these bodies, as to render its extraction difficult. [The cases are interesting, but the evidence of these bodies being organized is far from satisfactory.]

*Operative midwifery. Symphyseotomy.* Dr. David Smith* advocates this operation on theoretical grounds, and by reasoning which presents nothing novel, as a substitute for craniotomy in cases of contraction of the outlet of the pelvis too considerable for delivery to be effected by the forceps, and yet where a comparatively slight increase of room would allow of the passage of the child. [The arguments by which it has been attempted to support this operation, are most ably refuted by Michell, De Synchondromonia Pubis, 8vo, Amstelod, 1783; see also the remarks of Kilian in his Operationslehre, Bd. ii, p. 867; and the statistics of the operation as given in Churchill's Operative Midwifery, p. 247; all of which furnish unanswerable objections to its performance under any circumstances.]

*The Cæsarean section.* References are given below to seven cases† in which this operation was performed with complete success; the life both of mother and child having been preserved. In Dr. Mestenhauer's case, the operation had already been performed once before in consequence of the presence of a large bony tumour in the pelvis. M. Aubinais‡ relates a case in which the life of the mother, and Dr. Etlinger§ another, in which the life of the child was preserved. Six cases are related in which both mother and child were lost.‖ [The first of these cases is a remarkable instance of that unhappy procrastination to which it is in great measure owing that the results of the Cæsarean section in this country are so almost invariably fatal. The statistics of the operation, including the cases collected by Kayser in his valuable dissertation which contains none but well-authenticated cases are as follows: The operation has been performed 364 times; in 139 the women recovered, in 225 they died; or the recoveries were in the proportion of 38 per cent.; or as 1 to 2·6. The fate of the child is stated in 304 instances; in 209 it was saved, in 95 it died, or 2 out of 3 children were saved.]

*Premature labour.* Professor Hoffman¶ has published a very elaborate and learned defence of this operation; which will possess greater interest on the continent than in our own country where the operation has so long been approved of, and practised. Dr. Simpson** advocates the induction of premature labour in cases where the death of the fetus has been found to occur frequently during the latter months of utero-gestation. [This practice is propounded by him as if it were novel, but it apparently had escaped his memory that Denman has practised and recommended it under the same circumstances.] Three cases†† are recorded of attempts to induce premature labour, by the use of the

* Northern Journal of Medicine, Jan. 1845.

† Dr. Ziehl, Bull. Gén. de Thérap. Février 1844; Dr. Luzzani, Il Filiatre Sebezio, Sept. 1843; Dr. Mestenhauer, Oesterr. Med. Wochenschr. April 27, 1844; M. v. Thibault, Archives Gén. de Méd. June 1844; Dr. Bresciani di Borsa, Ibid. June 1845; Dr. Jehn, Casper's Wochenschr. April 12, 1845; Lebleu, Gaz. de Méd. Mai 10, 1845, p. 298.

‡ Gaz. Méd. Mai 10, 1845, p. 297.

§ Observationes Obstetricæ, etc. 4to; Bonnæ, 1844.

‖ Cox, Prov. Med. and Surg. Journal, Sept. 18, 1844; v. Thibault, Arch. Gén. de Médecine, June 1844; Etlinger, Op. cit. p. 49; Coley, a case of Cæsarean Operation, &c. Bridgnorth, 8vo; Löhr, extracted in London and Edinb. Monthly Journal, April 1845, p. 323; E. von Siebold, Neue Zeitschr. f. Geburtsk. Bd. xviii, 1 Heft, p. 45.

¶ Ibid. Bd. xv, Heft 3, and Bd. xvi, Heft 1.

** Lond. and Edinb. Monthly Journal, Feb. 1845.

†† By Drs. von Haselberg, Feldmann, and Naegele of Dortmund, in Preuss. Med. Zeitung, Jan. 10, April 10, Dec. 4, 1844.

vaginal tampon as employed by Schöller; but the results can scarcely be regarded as favorable to the proceeding since uterine action was not induced in the first case till after 40 hours, and in the third till 23 days, [during the whole of which time the child must go on increasing in size, and the advantages of the operation be daily diminishing.] Von D'Outrepont* prefers puncture of the membranes to any other mode of inducing premature labour; concerning which he has concluded after giving them a trial, that they are either uncertain in their action, or expose the mother to risk, or at least to pain and annoyance far exceeding that produced by puncture of the membranes, or else they peril the child still more. An interesting case is recorded by Drs. Hoeniger and Jacoby,† in which they employed galvanism to excite uterine action in a case where the introduction of sponge tents into the os uteri, and the administration of the ergot of rye had been had recourse to without effect, in order to induce premature labour. The use of an electro-magnetic apparatus was immediately followed by the reexcitement of uterine action. Its use was continued only for half-an-hour, at the end of which time the os uteri was so far open that the membranes could be easily ruptured, and in another half hour the child was born alive.

*Uterine hemorrhage.* M. Loir and Mr. Thompson,‡ both record a case of fatal internal hemorrhage, occurring before the birth of the child. In M. Loir's case, the symptoms of faintness, exhaustion, &c., occurred in the 7th month of pregnancy, and were almost unattended with uterine action. The os uteri was undilatable, and delivery was effected by incising it, and extracting the child. The patient soon died; and on a post-mortem examination the placenta was found detached at its centre by an immense effusion of blood between it and the uterus. Mr. Thompson's case closely resembles the preceding in the sudden accession of faintness, and the almost total absence of uterine action. The patient died undelivered, the placenta being completely detached from the uterus, and an immense effusion of blood having taken place between the membranes of the ovum and the womb. In neither case was there the slightest escape of blood externally. [Cases similar to the above have been collected by Baudelocque in his essay on this subject, and are referred to by Velpeau, Traité des Accouchemens, tome, ii, p. 88; a case is likewise mentioned by Mr. Crowfoot, in Ed. Med. Surg. Journal, Oct. 1824; and another by the late Dr. Ingleby in his Lectures.]

*Unavoidable hemorrhage.* Dr. Simpson,§ in a very interesting paper on this subject, has collected 141 cases of placenta presentation in which that body was either expelled or extracted before the child; only 10 of which were followed by the death of the mother, while 115 out of 399 cases of placenta previa, treated in the ordinary way proved fatal. It further appears that the presence or absence of flooding after complete separation of the placenta is not influenced by the length of time that elapses between its detachment and the birth of the child. It was apparently a knowledge of these facts, though not an acquaintance with their full extent, that induced Mr. Kinder Wood|| to recommend the complete detachment of the placenta in some cases of unavoidable hemorrhage. This practice has with certain modifications been advocated by Dr. Radford¶ of Manchester, and has likewise been followed by Dr. Simpson of Edinburgh, though he does not seem to have a fair claim to the honours of priority in its adoption. Dr. Radford aims to discountenance the too great haste in resorting to

* Neue Zeitschrift f. Geburtsk. Bd. xvi, Heft 1. † Ibid. Heft 3.

‡ Révue Médicale, Août 1844; Med. Gazette, Nov. 29, 1844.

§ London and Edinburgh Monthly Journal, March 1845. To these cases may be added another similar one by Mr. Tennent, Lond. and Edinb. Monthly Journal, June 1845.

|| Extracts from Mr. Wood's lectures, and copies of some of his cases are given by Dr. Radford, in Prov. Med. and Surg. Journal, Feb. 26, 1845.

¶ Provincial Medical and Surgical Journal, Dec. 24, 1844, and Jan. 22, 1845.

artificial delivery in all cases of hemorrhage, and to point out galvanism as a powerful agent in inducing uterine contraction. His recommendation is not merely theoretical, but he has employed it successfully in one instance by applying one conductor of an electro-magnetic apparatus to the os, and the other over the fundus uteri; and breaking contact occasionally, but continuing to employ it till the desired effect was produced. Galvanism has since been used with success in a case of uterine hemorrhage by Mr. Cleveland.* [The employment of galvanism to excite uterine action had already been suggested by v. Herder in his Beiträge zur Erweiterung der Geburtshülfe, Leipsig, 1803; and Stein had with the same view recommended the use of forceps, the two blades of which should be of different metals,† but Drs. Hoeniger and Jacoby, and Dr. Radford are unquestionably the first who have reduced the suggestion to practice.] Dr. Radford cautions against attempts to deliver in cases of unavoidable hemorrhage, before the os uteri is sufficiently dilated. He recommends the rupture of the membranes in cases of partial placenta presentation, advises when the placenta is seated fully over the os if exhaustion be present, that the liquor amnii be drawn off gradually by perforating the placenta, (with an instrument like that of Holmes for the induction of premature labour,) and that the placenta be then completely separated. He further advocates the detachment of the placenta, coupled when necessary with the use of galvanism as generally applicable to all cases of complete placenta presentation.

*Midwifery statistics, reports of hospitals, &c.* The note contains references to papers on these subjects.‡ Dr. Ramsbotham's tables are very valuable, since they embody the results of 35,743 deliveries; and an account of about half that number forms the subject of Professor Klein's report. The mortality of the hospital at Vienna as stated by Dr. Klein, amounted to 6·6 per cent.; while that of the Dublin Lying-in Hospital under Dr. Collins, was 1 per cent., and of the patients of the London Maternity Charity ·4 per cent. Some explanation of this occurrence may be thought to be afforded by the fact that Dr. Klein used the forceps once in 32 times, Collins once in 608, and Ramsbotham once in 729. The actual mortality among the forceps cases does not appear from Dr. Klein's report.

### THE PUERPERAL STATE.

*Puerperal fever.* The learned work of Dr. Litzmann,§ which contains a historical sketch of the principal epidemics of this disease that have prevailed at any time, supplies a deficiency in medical literature. Dr. Litzmann likewise describes an epidemic which he witnessed at Halle in the years 1840-1. Dr. M'Clintock has detailed|| the particulars, an epidemic which broke out quite unexpectedly at the Dublin Lying-in Hospital, in the early part of 1845. This epidemic was characterized by great depression of the vital powers, and proved speedily fatal. During the time of its prevalence, erysipelas was unusually frequent in other hospitals in the city. Dr. Blackmore¶ has published a series of papers, the object of which is to establish the identity of the poison

* Medical Gazette, June 27, 1845.

† Busch und Moser's Handbuch der Geburtskunde, Band ii, Art. Galvanismus.

‡ Dr. H. F. Ramsbotham, report of the Maternity Charity, Med. Gazette, May, June, July, 1844, and in the Appendix to his book; Dr. Burwell, report of the Philadelphia Hospital, Amer. Journal of Med. Science, April 1844; Dr. Murphy, report of Midwifery Practice at University College, Dublin Journal, Nov. 1844; Dr. W. Campbell, statistics of 5754 cases, in Northern Journ. of Med. June 1845; Mr. Watson and Dr. Waddy, Prov. Med. and Surg. Journal, Dec. 4, 1844, and Jan. 15, 1845, cases in private practice; Professor Klein, report of Lying-in Hospital at Vienna, Oesterr. Med. Jahrb. Jan., Feb., März 1845.

§ Das Kindbettfieber, etc.; Halle, 1844, 8vo.

|| Dublin Journal, May 1845.

¶ In the Prov. Med. and Surg. Journal during the early part of 1845.

of puerperal fever with that of erysipelas, and to show that the treatment applicable to it is not such as would be suitable in cases of sthenic inflammation. Mr. Storr,* of Doncaster, relates several cases in illustration of the fact that the contagion of puerperal fever may produce in persons not in the puerperal state, either peritonitis, or inflammation of some of the serous membranes attended with low fever; or local or general erysipelas, or various forms of typhus fever. A remarkable illustration of the affinity between puerperal fever, and other diseases the result of morbid poisons is contained in Dr. Murphy's Report of the Obstetric Practice of University College.† Having had occasion to remove the placenta of a patient who died soon afterwards from puerperal fever, several pustules appeared two days afterwards on the arm which he had introduced into the uterus. The appearance of these pustules was attended with considerable constitutional disturbance, and one of them assumed much of the character of malignant pustule. Mr. Farr‡ has made some observations on the rate of mortality in childbed, and on the share which puerperal fever has in its production. He has likewise published in his report an interesting document furnished by Mr. Bossey of Woolwich, which shows by facts occurring in his own practice, the highly contagious nature of puerperal fever. M. Botrel§ describes two epidemics of puerperal fever which prevailed in the city and hospital of Rennes, in 1842 and 1844. He proposes for the disease as he observed it, the name of angioleucite uterine, since it was characterized by inflammation of the uterine lymphatics without any affection of the veins. The blood presented various deviations from a healthy condition, and purulent deposits in the lungs were by no means unusual. He believes that it depended on atmospheric causes, especially on dampness and highly electric conditions of the air, but rejects, though scarcely on adequate grounds, the influence of deficient ventilation in its production as well as the notion of its being propagated by contagion. Its attacks usually commenced with violent febrile symptoms, soon followed by a condition of stupor. The abdominal pain, at first confined to the uterine region, extended speedily over the whole abdomen, and for a short period was very excruciating, but ceased almost entirely as the state of depression increased. The prostration of all the powers, and the affection of almost all the functions of the body when this typhoid state supervened were very remarkable, and terminated after a short period in death. Some patients died in 36, others in 40 hours, but the 5th day was the period of the greatest mortality. Very few of those who were attacked survived; in 1842, only 4 recovered out of 24 who were attacked, and in 1844 only 2 out of 22. In those cases in which recovery took place local and general bleeding, purgatives, and mercurials were employed, and when these remedies failed to do good, all other means proved perfectly useless.

M. Marchal de Calvi‖ has published a very elaborate essay on *intra-pelvic abscess.* He treats of the disease in both sexes, but of the 75 cases he records 52 have reference to puerperal women. Of these 52 cases, 44 have already appeared in print, 8 have been communicated to M. Marchal by M. Bouchut, but none have fallen under his own observation. The chief value of his pamphlet consists therefore, in the care with which he has collected almost all recorded cases of this affection. Dr Lever¶ has written a paper on the same affection, containing the account of several cases that came under his own notice. He proposes for it the name of *pelvic inflammation,* in preference to any more definite designation, in consideration of the difficulty that there is in the way of determining what part is primarily affected, whether the cellular tissue of the pelvis or the uterine appendages. A case of this kind is likewise de-

* Prov. Med. and Surg. Journal, May 7, 1845. † Op. cit. p. 47.
‡ Fifth Report of the Registrar-general, pp. 380-96. § Arch. Gén. de Méd., Avril, Mai, Juin, 1845.
‖ Reprinted from the Annales de la Chirurgie, for 1844. ¶ Guy's Hospital Reports, 1844.

tailed by M. Fouquier,* and some of M. Simm's† observations on abscesses and chronic engorgements of the iliac fossa belong to the same category, though his remarks apply chiefly to the affection when it occurs independent of labour, and as one of the sequelæ of inflammation of the cæcum. [These essays confirm without adding anything material to the observations of Drs. Doherty and Churchill, referred to in the last Report.]

*Lactation*. Dr. Witte‡ has made some valuable practical remarks on the management of the breast during pregnancy, in order to fit it for suckling. In cases where the nipple is imperfectly developed, he recommends that, after it has been brought into a state of erection, by the application of a warm sponge, an apparatus should be employed consisting of a wooden nipple-shield perforated at its apex, and fitted to an Indian rubber bottle, while its inner surface is rendered adhesive by the application of some adhesive plaster. This is adapted to the nipple when in a state of erection, and the pressure of the hand being removed from the bottle a vacuum is at once formed, by which the nipple is gradually elongated as surely as by an ordinary breast-pump, and with less discomfort. The employment of this apparatus must be continued for five or ten minutes daily, during a longer or shorter period according to the state of the nipple.

Mr. H. H. Davies§ relates an instance of *supernumerary nipple* below the left breast, of the existence of which the patient was not aware until her fifth pregnancy. Dr. Chowne‖ likewise mentions a similar case in which the supernumerary nipple was situated two inches below the other nipple, and states that the same peculiarity had existed in this person's mother. He describes also a case of supernumerary mamma which was situated on the thigh, and until the occurrence of pregnancy had been supposed to be merely a nævus, but it then enlarged to the size of half an orange.

A remarkable case of *galactorrhea* is related by Dr. Green,¶ as having occurred in a lady aged 47 ; the mother of 4 children, of whom the eldest was born when she was 20, the youngest when she was 33. Ever since the birth of her first child the secretion of milk had continued unabated, but subject to increase at her menstrual periods. She had suckled her own children and two others, all of whom throve at the breast, and her own health had been unimpaired by the continuance of the secretion.

The appendix to Dr. Ashwell's work on Diseases of Women,** contains a very valuable essay on the morbid consequences of *undue lactation,* in which he shows that the symptoms resulting from it, though usually not appearing till after several months occasionally occur in the course of a few weeks ; that organic lesions may, though very rarely, result from undue suckling ; and that weaning the child is absolutely essential to the cure.

### II. On the Progress of Knowledge with Reference to the Diseases of Women.

Since the publication of the former Report, the concluding part of Dr. Ashwell's valuable work on this class of diseases has appeared.†† It includes the diseases of the lining membrane of the uterus, polypus, and displacements of the womb, as well as the diseases of the ovaries and of the external organs. Three out of four parts of M. Meissner's‡‡ work on the Diseases of Women have been published. It is written on the same plan as his manual on the diseases of children, and has all the merits of an extremely well-executed

* Bull. Gén. de la Thérap. Fevrier 1844.
† Ibid.
‡ Neue Zeitschrift f. Geburtskunde, Bd. xvi, Heft i, s. 75.
§ Med. Gazette, Jan 26, 1844.
‖ Lancet, June 15, 1844.
¶ New York Journal of Med. Sept. 1844.
** Part III, p. 721.
†† On the Diseases peculiar to Women, Part III, 1844, 8vo.
‡‡ Die Frauenzimmerkrankheiten; Leipsig, 1844-5, 8vo.

compilation, made by a man whose own opportunities for observation have been very considerable. Professor Kiwisch* whose work on Puerperal Fever was noticed in this Journal some years ago, has announced a work on the diseases of the unimpregnated state. The first volume on diseases of the uterus has appeared in Germany, but has not yet reached this country. Dr. Meigs has published a translation with notes, of the laborious compilation of M. Colombat de l'Isère.† A series of reports on the diseases of women have appeared from the pen of Dr. Rigby;‡ and Dr. Heming§ has contributed several essays on the same subjects.

DISORDERS OF MENSTRUATION.

*Amenorrhea.* Dr. Toogood‖ has published some extremely interesting cases in which fatal affection of the brain occurred in chlorotic patients, associated with suspension or irregular performance of the menstrual function. [Notwithstanding its popular title, the pamphlet will well repay an attentive perusal. It may be doubted indeed whether medical practitioners will not benefit by reading it more than the public to whom it is addressed.]

*Dysmenorrhea.* Dr. Rigby¶ has written a work in which he endeavours to apply the theories of Dr. Prout in explanation of some forms of painful menstruation. His treatise consists of two parts: in the former of which he notices the general results of derangement of the process of assimilation. He insists on the fact that the mucous membranes form one of the grand emunctories for the albuminous principle, when redundant or imperfectly assimilated. Hence it follows that disorders of the assimilative process determine corresponding disorder of the functions of the mucous membranes. Among these the uterine mucous membrane bears its part, and it is especially in connexion with the gouty or rheumatic diathesis that disturbance of its function is most frequent. These disorders are either active when like rheumatic affections of other parts, they are associated with congestion, and are attended by inflammatory symptoms, giving rise to dysmenorrhea; or chronic, attended with leucorrhea and chronic affection of the cervix uteri. Symptoms of general impairment of the digestive powers attend the affection, with excess of lithates in the urine, and the formation of fibrinous membranes by the uterus and painful menstruation, or with leucorrhea and chronic inflammation of the cervix uteri; inducing a gradual suppression of the menstrual flux. Five cases are related in illustration of the author's views respecting the disease, which he conceives is to be treated by remedies applied to the constitutional disorder rather than to the local ailment.

Dr. Simpson** has invented small bougies of German silver about 2¼ inches long, which he attaches to a temporary handle, and introduces them into the os uteri in cases of dysmenorrhea connected with stricture of the orifice of the womb. After being introduced, the handle is unscrewed, and the bougies are left for two or three days. They are more convenient and are said to cause less annoyance than ordinary bougies which were employed for this purpose by the late Dr. Mackintosh.

*Menorrhagia.* M. Ginestet†† praises highly the expressed juice of the common nettle, *urtica dioica,* which he gave successfully in ℥ss doses in an obsti-

* Klinische Vorträge ueber specielle Pathologie und Therapie der Krankheiten des weiblichen Geschlechts; Prag. 1845, 8vo.

† A Treatise on the Diseases and Special Hygiene of Females, by C. de l'Isère. Translated with additions, by C. D. Meigs, M.D. Philadelphia, 1845, 8vo.

‡ In the Medical Times for 1844-5, passim. § In the Lancet for 1845, passim.

‖ Hints to Mothers, and other persons interested in the Management of Females at the age of Puberty; London, 1844, 8vo, pp. 20.

¶ On Dysmenorrhea, and other uterine affections in connexion with derangement of the assimilating functions; London, 1844, 8vo.

** Lond. and Edinb. Monthly Journ., Aug. 1844. †† Bull. de l'Acad. Roy. de Méd. Fevr. 28, 1845.

nate case of menorrhagia. [His recommendation of the remedy appears, however, to be founded on the results obtained in a single case.]

*Discharges vicarious of menstruation, &c.* M. Fourget* relates the history of a patient aged 17, who having menstruated regularly for a year, had on one occasion hemorrhage from the skin of the face, the conjunctiva, and the mucous membrane of the mouth, vicarious of the natural function. This anomaly, however, occurred but once, the menses reappearing on the next occasion by the natural channel. Professor v. d'Outrepont† met with a woman who having for some years menstruated regularly injured her arm while menstruating. Ever since that occurrence, discharge of blood has taken place from this wound synchronously with the natural menstrual flux, and ceasing as that did when the patient became pregnant. MM. von Muynck and Kluyskens‡ detail the history of a woman whose menses having become scanty and attended with indisposition at each period, at length ceased at the age 52, but were immediately succeeded by a discharge of blood from the left nipple, which continued to return with regularity every month, until the patient's death at the age of 57½. M. le Conte§ relates a singular case in which a negress, aged 70, who had not menstruated for 20 years, began to do so after being struck by lightning, and had continued to menstruate regularly at the time of his report, twelve months afterwards.

### DISEASES OF THE UTERUS.

*Means of investigating them.* Various modifications of the speculum have been proposed by Dr. Warden, Mr. Smith, Dr. Dixon, and Mr. Moss.|| Dr. Warden has suggested the application of the reflective prism to specula for examining the ear, vagina, or other closed passages of the body. His very ingenious contrivance cannot be understood without minute description. He suggests moreover¶ a change in the ordinary cylindrical speculum which he conceives would secure most of the advantages to be obtained by the more complex instrument. This modification consists in the removal of an oblique slice from the end of the speculum, which would allow of a lateral view of the vaginal walls, and prevent that puckering of the mucous membrane, when it is introduced or withdrawn, by which a distinct view is often so much impeded. Mr. Smith's speculum is almost the same as Dubois' modification of Récamier's speculum, who had an aperture made in the cylinder for the purpose of detecting small vesico-vaginal fistulæ. Dr. Dixon's and Mr. Moss' specula so closely resemble each other, that in reality they are the same instrument. They consist of wire rods inserted at one end into a ring, and terminating at the other in probe-pointed extremities ; and furnished with a plug of polished wood, grooved so as to receive the wires Dr. Dixon's speculum is furnished with a contrivance for expanding it, which does not appear to exist in that invented by Mr. Moss. [These instruments may be serviceable in some operations on the vagina, but as specula their value must be very small.]

Dr. Simpson** suggests the employment of sponge tents to dilate the os uteri in order to allow of the introduction of the finger to ascertain the state of the cavity in cases of uterine disease; [but it may be doubted whether the uterus would often tolerate their presence, and likewise whether under ordinary circumstances such a dilatation of the os uteri as he speaks of, would be likely to result from their employment.]

*Displacement of the uterus. Prolapsus uteri.* Dr. Chaumet†† describes a modification of Dieffenbach's operation for the cure of prolapsus, which he

* Gaz. des Hôpitaux, Sept. 24, 1844.
† Neue Zeitschr. f. Geburtsk. Bd. xvi, Heft i.
‡ Oest. Med. Wochenschr. Dec. 14, 1844.
§ New York Journal of Med. Nov. 1844.
|| Medical Gazette, May 24, 1844; Ibid. July 5, 1844; Boston Med. and Surg. Journal, March 1844; Dublin Medical Press, April 16, 1845.
¶ Lond. and Edinb. Monthly Journ. Dec. 1844.
** Ibid. Aug. 1844.
†† Bull. de l'Acad. Roy. de Méd. Mars 15, 1845, p. 442.

adopted with success. This modification consisted in bringing the edges of the wound together by the interrupted suture, after removing a strip of vaginal mucous membrane $1\frac{1}{6}$ inches broad. The contraction of the vagina thus produced was very considerable, and the cure was permanent. Professor Blasius* describes a new operation for procidentia of the uterus, which consisted in the insertion of four circular ligatures beneath the mucous membrane of the vagina, bringing them out again into the passage, then reintroducing them under the mucous membrane, and thus causing them to surround the whole vagina. In the case which he relates the inflammation excited by these ligatures was sufficient to produce a contraction of the whole passage, such as retained the replaced uterus in its proper position, where it continued at the end of eight weeks. Dr. Toogood† relates the particulars of a case of procidentia of the uterus of long standing, in a lady aged 60; in which the organ having descended 7 or 8 inches beyond the external parts, and being quite irreducible, he applied a ligature round it, and then cut it away. It weighed two pounds, its cavity was obliterated, and its substance had acquired an almost cartilaginous hardness. The patient whose health was previously much impaired, recovered perfectly.

*Retroversion of the uterus.* Cases of this accident occurring in the unimpregnated state, are related by Dr. v. Kiwisch, Dr. Helmuth, Mr. Whitehead, and Professor Trefurt.‡ In Dr. v. Kiwisch's case, the displacement was produced by the pressure of a cyst which was most probably ovarian. The cyst suddenly burst into the rectum, whereupon the uterus returned to its natural position. In the second case it seems to have come on spontaneously. Only a week elapsed from the occurrence of the first symptoms to the supervention of complete retroversion. Attempts to effect reposition did not succeed, and were followed by an attack of uterine inflammation. As this subsided after rather active treatment the uterus began to return to its natural position, which at length it completely regained. Mr. Whitehead's case presents many instructive features. The uterus became retroverted in the first pregnancy, but the accident was remedied, and the patient was confined at the full period. The same accident occurred in the succeeding pregnancy, and was followed by abortion in the 3d month, and on two subsequent occasions the patient aborted at the same period. After each abortion she had the sensation of the womb not being in its proper place, and when Mr. Whitehead became aware of the real nature of the case, all attempts at reduction failed. The patient had been for some considerable time under medical care, before the real nature of her ailment was discovered, owing to an examination per rectum having been omitted. [It is probable that the abortions and the irreducibility of the retroverted uterus depended on the organ having contracted adhesions with surrounding parts, as described by Madame Boivin in her Recherches sur une des causes de l'Avortement.] The case related by Professor Trefurt is interesting, as having presented all the symptoms of retroversion in a very marked degree. The displacement appears to have existed for five years; ever since the birth of the patient's only child. No attempt at reposition could be made till after local depletion and other preliminary treatment had been adopted. It was then found impossible to exert any force on the misplaced uterus, without exciting most excruciating pain. A peculiarly-constructed pessary, invented by Dr. Sander, of Brunswick, and called by him *mochlo-pessum,* or lever-pessary, was introduced, and eventually tolerated by the patient. At the end of two months the uterus had become more moveable, and was more nearly approximated to its natural situation, and in a few weeks more the misplacement was completely removed. [This instrument of which a description and drawing are

* Med. Zeitung, Oct. 9, 1844. † Prov. Med. and Surg. Journ. July 10, 1844.

‡ Oesterr. Med. Jahrb. Feb. 1844; Casper's Wochenschr. Oct. 5, 1844; Med. Gazette, Sept. 13, 1844; and Op. cit. p. 280.

given by Kilian, in his Operationslehre, (Bd. iii, Taf. 3, fig. 13,) consists of a ring-pessary to which is attached a curved stem surmounted by an oblong cushion or pessary. A hinge at the origin of the stem allows its inclination to be varied, while a screw at its other extremity, renders it possible to vary its length, and consequently to regulate the pressure in an upward direction which the instrument exercises.]

*Inversion of the uterus.* Mr. Crosse's essay already referred to, contains a most interesting collection of facts illustrating the somewhat obscure subject of chronic inversion of the uterus, after parturition, or occurring in the unimpregnated state in consequence of the presence of uterine polypus, or of the influence of other similar causes. Dr. Oldham* describes and delineates a preparation of partial inversion of the uterus produced by a polypus which grew from the right side of the fundus uteri, and several most instructive diagrams and plates illustrative of this occurrence are given in Mr. Crosse's essay. Dr. Oldham's remark is doubtless correct, when he says that the inversion is the result of the action of the womb in its efforts to expel the polypus rather than of the mere weight of the body. Dr. Meigs† makes mention of two cases in which he believes that an inverted uterus became spontaneously restored to its natural condition. In one case the uterus was ascertained to be inverted five weeks after delivery, in the other this state had persisted for more than two years after the birth of the patient's child, notwithstanding which, both the women subsequently became pregnant. [It is easier, however, to conceive that even an experienced man should commit an error of diagnosis, than to understand how any efforts of nature could cure a chronic inversion of the womb.] Mr. Crosse, Dr. Esselman, M. Velpeau, and Dr. M'Clintock relate cases in which the inverted uterus was removed, and all the patients recovered with the exception of the woman operated on by M. Velpeau, who died of peritonitis, which supervened almost immediately after the operation. The person whose history is recorded by Dr. M'Clintock, is the fifth on whom Dr. Johnson of Dublin, has successfully operated. Two of his cases are related in vol. iii of the Dublin Hospital Reports ; the outline of two others is given in Dr. M'Clintock's paper, besides the particulars of the one already referred to, and the history of another woman whose health was too bad to allow of the operation being performed, and who died nine months after the occurrence of the accident, worn out by hemorrhage and mucous discharge from the uterus.

M. Tessier‡ adduces cases in proof of the existence of *dropsy* and *tympany* of the unimpregnated uterus, in reply to the assertions of MM. Stolz and Naegele, that such diseases are impossible. Their denial of the possibility of such occurrences in the unimpregnated state was founded on the fact of the lining of the membrane of the uterus being mucous, not serous, on its tissue being incapable of any distension, such as the occurrence of these diseases must imply, on the absence of any cause adequate to close the cervix, and on the non-existence of any authentic observations of physometra, or hydrometra. Besides detailing observations by various writers in support of his opinion, M. Tessier relates the particulars of an indubitable and very interesting case of tympanites uteri that came under his own notice.

*Inflammation and ulceration of the os and cervix uteri.* In a series of papers, which have since been republished in a separate form, Dr. H. Bennet§ gives the results of a series of observations on this subject, made at the Hôpital St. Louis, and other hospitals of Paris. He treats, first, of the affection as it occurs in women who have never borne children ; then in those who have been

* Guy's Hospital Reports, new series, vol. ii, pp. 105-36. † Op. cit. p. 183.

† Gaz. Méd. Jan. 5, 1844.

‡ In the Lancet during the spring of 1845, and under the title of A Practical Treatise on Inflammation, Ulceration, &c. of the Neck of the Uterus.

mothers, or are pregnant, and shows that in the latter circumstances the liability of the uterus, and especially of its cervix, to become the seat of morbid action is greatly increased. He differs from those who regard ulceration of the cervix as generally a secondary result, the mere attendant on, or consequence of, inflammation of the lining membrane of the organ, or of hypertrophy, or induration of the cervix. He conceives inflammation and ulceration of the cervix to be frequent as primary ailments, almost invariably present in cases of confirmed leucorrhea, very common as sequelæ of abortion, and by no means unusual after delivery at the full time, and that hypertrophy and induration are their consequences. In women who are virgins the inflammation and ulceration seldom extend deeper than the mucous membrane; but in those who have been pregnant the more deeply-seated tissues are often involved. He relates many cases of simple ulceration of the cervix uteri, describes its symptoms clearly, and insists on the necessity of the use of the speculum in many cases in order to detect its existence. The treatment which he recommends is chiefly local, consisting in the employment of astringents and caustics of various kinds, the merits of which he discusses fully. In the second part of his treatise he examines the subject of syphilitic and malignant ulcerations of the os and cervix uteri. M. Pareira* describes as a new and hitherto unnoticed uterine disease, the formation of an adventitious fibrous tissue on the cervix uteri and parietes of the vagina. He states that in some cases of long continued and intractable uterine disease, he has found the cervix uteri drawn from its natural position, and tied down to the walls of the vagina by a kind of false membrane composed of fibrous tissue, or that bands of a similar structure are sometimes found running along the parietes of the vagina. [The description that he gives is very obscure; but in all the cases which he describes inflammation of the vagina and ulceration of the cervix uteri either were present or had existed, so that these bands were in all probability the cicatrices which are known to result sometimes from inflammation in those situations, and no new and hitherto unknown disease.]

M. Estevenet† relates the very extraordinary case of a woman who, having suffered for two years from uterine symptoms, discharged a large mass per vaginam, which was ascertained to be the whole *body of the uterus* without its appendages, which had been *detached* from its connexions *by a slow process of inflammation.* The woman died on the eleventh day. [The anatomical details are very minutely and carefully recorded, and there seems to be no reason to doubt the correctness of the observation.]

*Polypus uteri.* Dr. Cambernon‡ proposes the strange theory that uterine polypi and fibrous tumours of the uterus result from the entanglement of ova in the uterine tissue, and their undergoing a kind of morbid development in that situation. The correspondence of the age at which these affections most frequently occur with that during which the reproductive system is in a state of activity, and their comparative rarity in virgins are the only facts on which Dr. Cambernon's hypothesis is grounded. Dr. Oldham§ makes some remarks on the mode in which polypi are supplied with blood, and on the source of the hemorrhage which they occasion. He distinguishes two sources of this hemorrhage: the discharge of blood taking place in some cases from the pedicle, in others from the surface of the tumour. He regards the former as the source of the hemorrhage in those cases where the polypus is a fibrous tumour of the uterus pediculated, while bleedings from the surface occur in the cellular or fibro-cellular varieties. Polypi of the former kind derive their supply of blood almost exclusively from that portion of the uterine tissue which forms their investment, and from which arterial trunks proceed,

* Gaz. Méd. Aug. 31, 1844.

† Bull. de l'Acad. Roy. de Méd. Aug. 31, 1844.

‡ Gaz. Méd. Février 1844.

and penetrate their substance. The veins, however, though closely collected around these growths, and presenting a considerable increase of size, do not penetrate their substance, and do not extend beyond their pedicle. The fibro-cellular variety, however, presents a very different arrangement; the cells observed in the interior of these polypi being, in Dr. Oldham's opinion, truncated and divided veins, which traverse the whole tumour, freely communicating with each other, and forming a very extensive venous circulation. [It is by no means certain, however, that these canals are venous; the contrary opinion entertained by Meissner, (see his Frauenkrankheiten, Bd. i. p. 833,) is much more probable, and the rather since these canals terminate by open extremities, an arrangement which, notwithstanding Dr. Oldham's revival of the old theory that the uterine veins terminate in a similar manner, is quite contrary to all that we at present know of the structure of the vascular system.] A case of spontaneous strangulation of a polypus by the os uteri, and its consequent detachment, is related by Dr. Garden.* M. Mayor† has twice successfully employed torsion, as recommended by M. Amussat, for the removal of two large uterine polypi; and in neither case did any ill result follow the proceeding. Various modification in the operation of tying polypi are suggested by MM. L. Boyer, Quackenbush, Bedingfield, and v. Watmann,‡ having for their object the easier and more certain application of the ligature. M. Boyer's instrument is rather complicated; the others are very simple, even more so than Gooch's canula; but a description of them would scarcely be intelligible unless accompanied with a drawing. M. Mollet§ relates the history of a woman who had suffered from symptoms of uterine disease for a year, when a tumour appeared beyond the vulva, and increased rapidly in size. It was supposed to be the inverted uterus, and under this supposition a ligature was applied on the fifth day, around the neck of the tumour, and it was then removed by the knife. It was now found that the extirpated uterus had not been inverted at all, but merely drawn down by an immense polypus attached to the os tincæ. The patient died on the fifth day after the operation.

*Fibrous tumours.* Dr. Roberts,|| in some remarks on these growths, confirms Dr. Ingleby's remarks with reference to the frequency of hysteralgia as a consequence of their presence. He notices that they have a peculiar predilection for the posterior wall of the uterus, and that though they may disappear under the use of medicine, they have a great tendency to return. He confirms Dr. Ashwell's observations with reference to the utility of iodine in their treatment. Dr. Pancoast and M. L. Boyer¶ have each removed a large fibrous tumour from the uterine cavity; in the former case the operation was attended with success, in the latter the patient died on the sixth day. In both instances the tumour was enucleated partly by the finger, partly by the help of instruments. In M. Boyer's case the tumour had not passed the os uteri, which it was necessary to incise, while in Dr. Pancoast's patient the tumour had passed out of the uterus, and nearly filled the vagina. The different result of the two operations seems to have been greatly dependent on the different relations of the tumours to the os uteri.

*Malignant diseases of the uterus.* Dr. Barbieri** relates a case in which an ill-constructed ring-pessary having been retained for several months, became imbedded in an ulceration of the cervix uteri, and gave rise to symptoms which were supposed to be those of ulcerated carcinoma. The presence of the pessary was not at first detected, but on the removal of the instrument the patient

* American Journal of Med. Science, April 1844.

† Gaz. Méd. Aug. 17, 1844.

‡ Bulletin de l'Acad. de Méd., Fév. 29, 1844; New York Journal, Jan. 1844; Lancet, May 11, 1844; Oesterr. Med. Jahrb., Jan. Feb. 1845.

§ Annales de Thérap., Janvier 1845.

|| New York Journal, Sept. 1844.

¶ Boston Med. Surg. Journal, Oct. 9, 1844; Revue Médicale, Mars 1845.

** Gaz. Med. di Milano, 29 Giugno, 1844.

speedily got well. Dr. Montgomery* has removed a cauliflower excrescence of the uterus by means of the ligature, and the patient continued well at the end of two months, having menstruated regularly during that period. He recommends the use of the ligature in the treatment of such cases, since the pain it occasions is comparatively slight, and there is no reason to fear serious hemorrhage after its application, while the good which results from it is almost immediate. He advises that after the separation of the part inclosed by the ligature the wounded surface should be at once touched with some powerful caustic. Dr. Riberi† has published an account of four cases of malignant disease in which he amputated the cervix uteri. All of the patients were under 40, and the disease, which was fungoid rather than pure scirrhus, was not of long standing, though extensive. The disease returned in every instance within three months after the operation, attended with profuse hemorrhage, and had already proved fatal to three of the patients at the time of his communication.

DISEASES OF THE UTERINE APPENDAGES.

*Diseases of the ovaria.* Dr. Kohlrausch‡ describes the anatomical structure of an *ovarian cyst containing hair and teeth.* It was made up of many cysts, some of which were filled with gelatinous matter. Others, which had thicker parietes, contained fat, hair, and teeth. The cysts were lined with flattened epithelium scales, below which was a layer of cells not flattened, then a structure resembling cutis, and below that a kind of sub-cutaneous cellular tissue. Hairs were implanted in a perfectly normal manner into this cutaneous tissue, in which there likewise existed greatly developed perspiratory and sebaceous follicles. The teeth were imbedded in a piece of bone in the wall of one of these cysts. They were all inclosed in tooth sacks, and in different stages of development.

Cases of the spontaneous rupture of *dropsical ovaria* are related by Dr. Fraser, Dr. Lambrecht, and M. Camus.§ In the first case the bursting of the sac into the rectum was followed by permanent cure; in the second the patient recovered after it had twice emptied itself at the navel; and in the third case the cyst burst on three occasions into the abdominal cavity, followed each time by the rapid absorption of the effused fluid, and great temporary improvement in the patient's condition. Mr. Brown,|| in two very interesting papers, combats the generally received opinion concerning the uselessness of medicine in ovarian dropsy, and relates five cases in which the plan which he advocates proved very successful. This plan consists in the internal employment of small doses of mercury, and in the use of mercurial frictions to the abdomen, so regulated as to keep the mouth slightly sore for some weeks; and in the administration of diuretics, succeeded by tonics, while the food is light and unstimulating, and daily exercise attended to. The local treatment consists in careful and constant tight bandaging the abdomen with flannel. When these measures appear to have taken effect, by the non-increase or positive decrease of the tumour, he advises that the cyst be then tapped and emptied. After the operation, pads should be applied over the cysts, and tight bandaging should be continued for three weeks, and the friction and medicines for at least six weeks longer. A case is related by Mr. Atkinson,¶ in which a woman, aged 53, was tapped 78 times in seven years and a half, six gallons being drawn off at each of the first 50 operations, but only half that quantity on each subsequent occasion. The interval between the operations, which used to be five months, came at last to be only three weeks, but the patient resumed her usual

* Dublin Journal, Jan. 1845. † Gazetta Medica di Milano, 9 Settembre, 1843.
‡ Müller's Archiv, 1843, Heft iv, p. 365.
§ Montreal Med. Gazette, May 1844; Med. Zeitung, Nro. 30, 1844; Revue Médicale, Nov. 1844.
|| Lancet, May 4, 1844, and April 5, 1845. ¶ Ibid. July 20, 1844.

active habits in a day or two after each puncture. Dr. Hamper* has recorded the history of a woman in whom ovarian dropsy came on after labour. The patient was treated by mercurial inunction, tight bandaging, and puncture of the cyst through the vagina, with complete success. Dr. Marshall Hall† has suggested that before resorting to any operative measures for the cure of an ovarian tumour, a probe be introduced and passed round it, so as to discover whether or no adhesions exist; also that an exploratory puncture should be made in order to ascertain the nature of its contents. Mr. Cazeaux,‡ in an essay on the treatment of ovarian dropsy, records a case in which permanent cure followed medical treatment and a single puncture of the cyst. He next relates the unsuccessful issue of an attempt to maintain a fistulous opening into an ovarian cyst, fatal peritonitis having been induced. A third instance is detailed in which death from peritonitis followed on puncture per vaginam, and an attempt to keep an elastic canula in the puncture. He states that puncture per vaginam has been performed twelve times; that the details are incomplete in three instances, that three of the patients died, that a relapse occurred once, and that five of the patients were completely cured. He lastly gives the particulars of another fatal case, in which death from peritonitis followed on a modification of the minor operation, the cyst having been only partially extracted, in consequence of the presence of some tumours, which rendered its complete removal impossible.

Four cases of the successful extirpation of the ovaria by the small incision are recorded by Dr. F. Bird, Dr. Emiliani, and Mr. Page,§ and three of a successful result following the major operation in the hands of Drs. Bowles, Clay, and Atlee.‖ In the case recorded by Dr. Emiliani, the operation had been performed by his father in the year 1815; the patient had since given birth to five children, two of whom were twins, and was in perfect health at the time of his writing the history of her case. Dr. Clay's patient was sufficiently recovered to return to her home, which was at a considerable distance, fifteen days after the operation. Dr. W. L. Atlee's patient had an attack of intense peritonitis immediately after the operation, but did well eventually. He assumes, in the history he has given, that the tumour was not ovarian, but that it grew from the surface of the uterus, and therefore calls the case one of extirpation of a fibrous tumour of the uterus. This idea did not strike him while performing the operation, but was an after thought [for which there does not appear to be any reasonable foundation.] Dr. J. L. Atlee¶ has published a full account of the case in which he successfully removed both ovaries, and to which reference was made in the last Report Only one case of failure of the operation has been recorded during the past 18 months. It occurred in the practice of Dr. W. L. Atlee,** the patient dying of peritonitis five days afterwards. Mr. Walne has likewise published the particulars of his two unsuccessful cases mentioned in the last Report. Dr. Churchill, Mr. B. Phillips, Dr. Jeaffreson, and Dr. W. L. Atlee†† have investigated the statistics of the operation, and have drawn up more or less elaborate illustrative tables. The chief results at which they have arrived are here thrown into a tabular form; the former table showing the rate of mortality from both operations in all cases where the extirpation of the ovary was either attempted or actually performed, and the latter the comparative mortality from the two operations in all cases in

* Oesterr. Med. Wochenschr. July 20, 1844. † Lancet, March 9, 1844.

‡ Annales de la Chirurgie, Oct. 1844.

§ Med. Gaz. March 22 and Aug. 16, 1844; Bulletino delle Scienze Mediche di Bologna. Dec. 1843; and Lancet, April 5, 1845.

‖ American Journal of Medical Sciences, January 1845, p. 255; Medical Times, Feb. 15-22, 1845; American Journal, April 1845.

¶ American Journal of Med. Sciences, Jan. 1844. Ibid. July 1844. ** Med. Gaz. Feb. 23, 1844.

†† Dublin Journal, July 1844; Medico-Chirurg. Transact. vol. xxvii, p. 468; Med. Gaz. Oct. 18, 1844; American Journal, April, 1845.

which the ovary was removed. Dr. Churchill takes 4 inches, and Mr. Phillips 6 inches as the line of distinction between the major and minor operations.

TABLE I.

| Authority. | No. of Cases. | Deaths. | Rate of Mortality. |
|---|---|---|---|
| Churchill | 66 | 24 | 1 in 2·75 or 36·3 per cent. |
| Phillips | 81 | 32 | 1 in 2·50 .. 39·5 .. |
| Jeaffreson | 74 | 24 | 1 in 3 .. 32·4 .. |
| Atlee | 101 | 38 | 1 in 2·65 .. 38· .. |

TABLE II.

| | *Major Operation.* | | *Minor Operation.* | |
|---|---|---|---|---|
| Authority. | No. of Cases. | Rate of Mortality. | Number of Cases. | Rate of Mortality. |
| Churchill | 34 | 38·2 per cent. | 15 | 13·3 per cent. |
| Phillips | 40 | 47 7 .. | 20 | 30· .. |
| Atlee | 75 | 41·2 .. | 18 | 27·7 .. |
| | Average | 42·3 .. | | 23·3 .. |

DISEASES OF THE VAGINA AND EXTERNAL ORGANS.

*Vesico-vaginal fistula.* Dr. Keith* relates the particulars of two cases of vesico-vaginal fistula of long standing, which were successfully treated by cauterization. The former of these cases is interesting, from the fact that the patient having attempted to close the aperture by a piece of cork, this cork slipped into the bladder where incrustations formed around it, and it constituted the nucleus of a calculus, which it was necessary to remove before treating the fistula. The foreign body in the bladder, however, had to a great extent served the part of a valve in blocking up the aperture, so that during a great part of the twelve months of its sojourn in that organ, the patient had passed her urine by the urethra, and the fistulous opening underwent during this period a very considerable diminution in size. Dr. Keith suggests whether in some cases of large vesico-vaginal fistula an Indian-rubber bag, filled with mercury might not be introduced through the opening into the bladder, where it might act as a valve, and thus favour the contraction of the fistula till it became small enough to cauterize, when the bag might be removed by crushing it and drawing it through the urethra. M. Lallemand† relates an instance of the complete closure of a small vesico-vaginal fistula which had existed for a year by means of the *sonde-airigne;* [see the previous Report, where this method is fully described.] In three other cases that came under his care during the same period this mode of treatment was ineffectual, and M. Serre states that of 15 patients treated in this manner by Prof. Lallemand, 5 died, 3 were made worse, 5 were left in precisely the same condition as before treatment, and only 2 were perfectly cured. Mr. Harrison‡ has described an instance of the successful palliative treatment of a small fistula, by introducing a skein of six threads of silk along the urethra, and through the fistulous aperture. These threads were removed one by one; the fistula having become almost closed; but the patient has never been able to dispense with employing one thread, which she changes every month. Using this precaution, however, not a drop of urine has escaped for the past three years, while for the five previous years she had been utterly unable to retain it. M. Béclard§ attempted to cure a bad case of vesico-vaginal fistula by obliterating the vulva, [as in the unsuccessful case of M. Vidal de Cassis.] For a time the operation appeared likely to succeed, union having been effected to a great extent; but peritonitis came on, which carried off the patient 17 days after the operation. In the discussion

* London and Edinburgh Monthly Journal, Jan. 1844, p. 13.

† L'Expérience, Sept. 12, 1844.

‡ Provincial Medical and Surgical Journal, June 11, 1845.

§ Bulletin de l'Acad. Roy. de Méd. Feb. 28 and March 15, 1845.

which followed the relation of this case, the operation was condemned by MM. Dubois, Gerdy, and Blandin, who considered that it leads only to a partial success, while its performance is exceedingly hazardous.

M. Deville* describes an affection almost peculiar to pregnant women, and which he calls *granular vaginitis,* from its resemblance to the granular metritis described by Boivin and Dugès. It is a disease of an essentially chronic nature, characterized by the development on the vagina of large, indolent red granulations, sometimes isolated, at other times confluent, occupying a varying extent of mucous membrane, and attended with an abundant, purulent, greenish discharge. The disease has always yielded to lotions of the nitrate of silver. M. Deville appears to be in error in supposing that this affection has been hitherto almost unnoticed, since it has already been described by M. Ricord† under the name of *psorelythrie.*

M. Desruelles‡ describes a peculiar affection of the vulva of which he has observed two cases, [and which appear to have approximated closely in their characters to that disease which has been described as *elephantiasis labiorum pudendi.* See Meissner, Frauenkrankheiten, Bd. i, p. 248.] The labia were of a pale blueish colour, soft in some parts, but presenting hard nodules in others, though the skin investing them was smooth. They were painless to the touch, as was the mons veneris, which was in a great measure denuded of hair, and presented a rugose mammillated surface, as though formed by flattened tubercles united at their base. The inguinal glands were of a stony hardness. In one case there was suspicion of a syphilitic taint and good appeared to result from the use of mercury, while in the other the disease was not benefited by that remedy.

Dr. Riberi§ relates with great minuteness the particulars of a case in which he extirpated the whole *urethra,* which he supposed to be *affected with scirrhus,* and which had been preternaturally enlarged ever since the patient's 11th year, she being 58 at the time of the operation. Doubt may be entertained as to the nature of the affection, but the minute account given of the difficulties met with in performing the operation cannot fail to be useful to any one who shall hereafter repeat it. It appears from M. Riberi's statement that notwithstanding the complete extirpation of the urethra, the patient could retain or void her urine at pleasure.

### III. On the Progress of Knowledge with Reference to the Diseases of Children.

### 1. diseases of the fœtus.

M. Hamel|| enumerates among the causes of the death of the fœtus, an *obese state of the umbilical cord,* by which probably he means the presence of an excessive quantity of the gelatine of Wharton. He conceives that this condition comes on about the fourth month of pregnancy, and that it proves fatal by compressing the vessels of the cord. He advises antiphlogistic treatment of the mother during pregnancy whenever on a former occasion the death of the fœtus has appeared to result from this cause. [The opinions of M. Hamel are by no means adequately supported in his essay by recorded facts.]

Dr. Cormack¶ has published a collection of all the cases in which *intra-uterine cystous disease of the kidney* has existed. He arranges the cases in three classes, according as they resulted from—1st, The presence of hydatid cysts; 2d, Obstruction of the ureters; 3d, The formation of cysts independent

* Arch. Gén. de Méd. July and August 1844.
† See remarks on M. Deville's essay in Gaz. des Hôp. Oct. 5, 1844.
‡ Arch. Gén. Mars 1844.
§ Gaz. des Hôpitaux, Feb. 25, 1845.
|| Bull. de l'Acad. Roy. de Méd. Feb. 28, 1845.
¶ London and Edinburgh Monthly Journal of Medical Sciences, Aug. 1844.

of either of the above causes. He describes and figures a case of this last variety which came under his own notice. In it a development of cysts takes place in the cortical substance of the kidney which communicate with each other, and thus form a multilocular pouch, while by their pressure they produce absorption of the renal substance. He believes that these cysts are formed by the abnormal development of the cells of the cellular tissue which unites the different parts of the kidney, and that they are consequently independent of hydatid disease, or of enlargement of the pelvis, infundibula, or tubular structure of the organ.

M. Sontag* has described and delineated a case of *congenital rhachitis* which occurred in the obstetric clinic at Heidelberg. [Schuetze's description and delineation of a similar case ought to have been mentioned in the last Report.† Other instances of this affection are recorded by Graetzer, Krankheiten des Fötus, p. 170.]

Dr. Simpson‡ communicates the particulars of two cases of *ichthyosis intra-uterina*—one of which, affecting the face only, was observed by Dr. Lewins; the other by Professor Vrolik. Professor Vrolik's views, with reference to the nature of the affection, coincide with those which had been previously expressed by Dr. Simpson. He thinks that the fissures of the skin are merely a secondary and mechanical result of the tegument not possessing a proper degree of expansibility, and not increasing with the growing dimensions of the fœtus. He conceives, moreover, that the general form of the child depends in these cases on arrest of development, to which as well as to the disease of the skin he refers the ectropium of the eyelids, and the peculiar form of the nose, mouth, and ears observed in these cases. A third case of the same disease is recorded by Dr. Smellie§, in which the child lived seven days, the diseased cuticle having begun to fall off on the second day, and having left the body nearly denuded of epidermis before death took place. Dr. Potts|| observed an instance of *congenital gangrene* of the left foot which caused death on the sixth day after birth. A line of demarcation between the sound and healthy parts formed on the second day above the ancle, and had exposed the bones when death took place. No cause could be assigned for the occurrence; it was not produced by the cord being twisted around the leg. Dr. Watson¶ describes a case of *congenital absence or ulceration of the skin* of part of the right leg and foot, which underwent a gradual process of cicatrization that was completed three weeks after birth. [The case was probably one of congenital deficiency of the skin, not of ulceration of a skin which had previously existed. For instances of this occurrence, see Graetzer, p. 231.]

Dr. Hedrich** relates the history of a woman who having been attacked by measles at the end of her pregnancy gave birth on the fourth day of the disease to a *female child* who was *covered with* the eruption of *measles*, and was suffering from catarrh, cough, sneezing, inflamed eyes, &c. but recovered in a few days.

### 2. GENERAL OBSERVATIONS ON THE MORTALITY AND DISEASES OF INFANCY AND CHILDHOOD.

*Infantile mortality.* Dr. Watt†† has published a series of tables to show that the proportions which deaths from certain diseases at given ages bear to the total amount of deaths from those diseases is the same in different cities and countries, though the actual amount of deaths from those diseases may differ very widely. Thus the mortality under 2 years of age, or under 5 years of

* Diss. inaug. pathologico-anatomica de rhachitide congenita; Heidelb. 1844.
† Symbolæ ad ossium recens natorum morbos, 4to; Berol. 1842.
‡ London and Edinburgh Monthly Journal, July 1844.
|| Northern Journal of Medicine, May 1844.
** Neue Zeitschr. f. Geburtsk. Bd. xv. p. 469.
§ Ibid. Dec. 1844.
¶ Ibid. Sept. 1844.
†† Lancet, June 8, 1844.

age from measles, scarlet fever, hooping-cough, &c. bears nearly the same proportion to the deaths from those diseases at all ages, in Glasgow, Edinburgh, New York, and Philadelphia. Mr. W. R Wylde* makes some observations on the diseases contributing most to infant mortality in Ireland; but the value of his conclusions appears to be much diminished by the probable inaccuracy of many of the returns with reference to the causes of death.

The appendix to the Sixth Report of the Registrar General contains much valuable information with reference to the rate of infantile mortality in the various countries of Europe. It appears from a communication by Dr. Fourgereaud,† that the mortality of children is very high at New Orleans, and that the most fatal disease there is the cholera infantum, since of 1106 deaths in infancy, 238 or 21 per cent. arose from this cause. It appears further, that while in Philadelphia there occurs one death from cholera infantum to 862 inhabitants, the proportion in New Orleans is as high as 1 to 375.

*Peculiarities of infantile diseases.* It is not possible to attempt to give an abstract of the very elaborate researches of M. Roger‡ on the *temperature in health and disease.* The following statements, however, embody some of the more important of his conclusions. Whenever the temperature exceeds 100°·4, whatever may be the state of the pulse and respiration, fever is present, either symptomatic or idiopathic. The temperature may rise as high in the course of local inflammation as in idiopathic fever; and the three diseases in which it reaches the highest point are typhus fever, pneumonia, and meningitis. A great elevation of temperature, as from 104° to 105°·8, when it coexists with but slight acceleration of the pulse, or a pulse not exceeding 100, may be regarded as pathognomonic of typhus fever. It is, however, not easy, especially in young children, to distinguish between enteritis and typhus fever, though if during a number of days the temperature have not been found to rise above 100°, or 102° the disease is probably enteritis; and typhus fever if it have exceeded that degree. Pneumonia may be inferred from a temperature of 104°, or 105°·8 if associated with acceleration of pulse and respiration, while in bronchitis the temperature does not exceed 104°. Diminution of temperature occurring in the interval between two periods of its increase is pathognomonic of meningitis; but in meningitis the temperature never rises so high as in pneumonia; the average maximum being about 102°. All the exanthemata are characterized by great increase of the animal heat, and great frequency of the pulse with moderate acceleration of the respiration. The temperature is highest, and this high temperature, averaging nearly 103°, is most sustained in scarlatina. In smallpox it averages 101°·8; but this temperature sinks after the commencement of the disease, until the stage of maturation, when it again rises, and a direct relation seems to exist between the danger to life and the height of the temperature. In measles the temperature, which on the average does not exceed 101°·2 is highest at the outset of the disease, and afterwards sinks progressively. It bears a direct proportion to the severity of the disease, and intensity of the eruption. In many diseases, such as dropsy, tubercle, hooping-cough, chorea, anemia, rickets, &c. the temperature is not changed. In some affections it is diminished, either partially, as in gangrene or paralysis, or generally, of which induration of the cellular tissue is a remarkable instance, since in that affection M Roger has found the temperature as low as 86°, and in one instance even as low as 71°·6.

Reference must likewise be made to the extremely good description given by M. Bouchut§ of the peculiarities which distinguish *febrile disturbance of the system in early infancy,* from similar affections occurring subsequently.

* Edinb. Med. and Surg. Journal, April 1845. † Boston Med. and Surg. Journ. Jan. 1844, p. 321.
‡ Archives Gén. de Méd. Juillet, Août, etc. 1844; Avril, Mai, etc. 1845.
§ Manuel Pratique des Maladies des Nouveaux-Nés, 12mo; Paris, 1845.

*Infantile therapeutics,* &c. Dr. J. B. Beck* has made some remarks on the influence of opium on the infant subject. His observations are for the most part judicious, and especially his advice that laudanum, tinc. camph. co., and Dover's powder, all of which are unvarying preparations, should be used in the treatment of children's diseases in preference to other opiates, such as the syrup of popies, the strength of which often varies. His remarks, however, apparently result from reading rather than from observation in actual practice. [His denunciation of opium is far too sweeping; and would tend, if acted upon, to deprive the practitioner of a valuable remedy; while no notice is taken of its different action in different diseases.]

Works have been published on the subject of children's diseases by M. Bouchut, Mr. Hood, and Dr. Condie, and M. Meissner's valuable hand-book has reached a second edition.† M. Bouchut's work has a more practical character than most French treatises on this subject, and may be regarded as an exposition of the opinions and practice of M. Trousseau. It treats exclusively of the diseases of infancy. Mr. Hood's is not a systematic treatise; but has been written with the object of showing that the share which inflammatory action has in the diseases of childhood has been much overrated, and consequently that antiphlogistic treatment is adopted much oftener than it should be. [This truth is however somewhat overstated, and the book is further remarkable for containing almost no allusion to the post-mortem appearances produced by those diseases the nature of which Mr. Hood discusses.] Dr. Condie's book is little more than a compilation in which the writer has contented himself with quoting the names of authors, without introducing references to their works.

### 3. DISEASES OF EARLY INFANCY.

*Asphyxia and apoplexia neonatorum.* In a paper which, notwithstanding its diffuseness and defective arrangement, contains much valuable matter, Dr. Doherty‡ investigates this subject. He justly objects to the employment of the term asphyxia to designate all varieties of apparent death in infants, the lungs being seldom the centre of the mischief, which in a large majority of cases has its origin in the brain. Of suspended animation from this cause there are two varieties: the first, which results from long-continued pressure on the head is attended with apoplectic symptoms and impairment of the respiratory process; but in the second, which occurs when the pressure on the head, is sudden and violent, the heart's action is immediately arrested. He points out the different treatment which these two conditions require, depletion being indicated in the former, but injurious in the latter. He next remarks that since in children stillborn the same *besoin de respirer* does not exist as in the adult whose respiration has been interrupted, there consequently is not the same necessity for the immediate employment of artificial respiration; but it is better to direct our first efforts to exciting the nervous energy. Death from apoplexy is one of the results of imperfect inflation of the lungs; and congestion of the surface, convulsions, and death, are occasionally noticed in cases where owing to this cause the heart's action has been irregular and tumultuous after birth. The death of the child, when the mother has died from hemorrhage, is owing to the placenta ceasing to oxygenate the blood of the child in consequence of the maternal veins being empty, and thus a kind of syncope of the fœtus is produced. When the child dies from pressure on the cord, if that pressure have not been continuous, the body presents the appearances of congestion; but if the interruption to the

* New York Journal of Medicine, Jan. 1844.

† Manuel Pratique des Maladies des Nouveaux-Nés, 12mo, Paris, 1845; Practical Observations on the Diseases most fatal to Children, 8vo, London, 1845; a Practical Treatise on the Diseases of Children, 8vo, Philadelphia, 1844; Die Kinderkrankheiten, etc. 8vo, Leipsig, 1845, 2 vols.

‡ Dublin Journal, March 1844.

circulation have been complete, the appearances observed are those of profound syncope, and the blood is found to have deserted the left cavities of the heart, and to be accumulated at its right side, and in the great venous trunks. In conclusion, he examines the effects of injury of the spinal cord of the child during labour, and gives illustrations of the resemblance between cases where that has occurred, and cases of trismus neonatorum. Dr. Walter* relates a case, apparently authentic, which shows how small is the need of respiration in the new-born infant. A woman gave birth to an illegitimate child, which, since it did not cry, she supposed to be dead, and buried it in a hole 18 inches deep. Over the child was a piece of coarse matting, and above this sand was strewn, though loosely, to the depth of 12 inches. In the cavern thus formed, and in which but a very small quantity of air could have been inclosed, the child was found alive at least six hours after its birth.

Dr. Fairbairn† has described the case of a female infant who died asphyxiated owing to retraction of the base of the tongue so far back into the pharynx, that it pressed on the epiglottis and closed the larynx. This accident was the result of congenital defect of the frænum linguæ, and malformation of the lower jaw. In a second case, a similar condition, though to a less extent, existed, and the child was reared by much care and constant watching.

*Cephalhæmatoma.* Dr. Hoffman‡ relates the particulars of a case in which having introduced his hand into the uterus for the purpose of turning a child whose side presented, he detected a cephalhæmatomatous tumour on the left parietal bone. A similar swelling likewise existed on the outer side of the right knee. This latter disappeared in a fortnight; the former was opened on the 12th day, and gave issue to thick blood, the wound healed in 16 days. [Though rare as a congenital affection, cases have nevertheless been observed, such cases are mentioned by Burchard, De Tumore cranii recens Natorum, p. 10; and by Graetzer, op. cit. p. 223.] Two cases of cephalhæmatoma are described by Mr. Adams,§ who has appended to this description an account of the affection compiled from different sources, but chiefly from the work of M. Valleix. The writer of this Report has given a description and drawing of a case of external and internal cephalhæmatoma complicated with fissure of the parietal bone, in which the child lived three weeks, and showed no sign of cerebral disturbance till forty-eight hours before its death in convulsions, although a very considerable quantity of blood had been poured out between the skull and dura mater, so as greatly to compress the right hemisphere of the brain. The case, however, was chiefly remarkable, in consequence of a reparative process having commenced on the interior of the skull, precisely similar to that which has often been observed on its outside in cases of external cephalhæmatoma. A bony ring had been formed around the tumour, and its surface was beginning to receive a bony investment by the deposit of numerous osseous plates, between the two layers of the dura mater. The fissure of the parietal bone could not be referred to any injury inflicted after birth, and though the labour had been natural, yet it appears probable that the injury of the bone must have occurred during parturition.||

A case of *facial hemiplegia*¶ in a new-born infant occurred in the clinique of M. Dubois, independently of any instrumental interference during labour. There existed, however, an osseous tumour in the pelvis of the mother, which may possibly have inflicted some injury on the child during labour, so that this case is probably no exception to the general rule, according to which facial paralysis in the new-born child is the result of mechanical violence.

*Trismus neonatorum.* Two cases of this affection, so uncommon in France,

* Neue Zeitschrift f. Geburtsk. Bd. xvi, p. 154.
† Northern Journ. of Med. March 1845, p. 278.
‡ Med. Zeitung, 31 Juli, 1844.
§ Northern Journal of Medicine, Dec. 1844.
|| Medico-Chirurgical Transactions, vol. xxviii.
¶ Gaz. des Hôp. Juin 3, 1845.

are related by M. Thore.* They present nothing remarkable in their history, except the fact that in one instance recovery took place, apparently in consequence of a most profuse bleeding from the bites of two leeches behind the ears, which caused a hemorrhage that was suppressed with great difficulty.

*Induration of the cellular tissue.* M. Roger's† observations on this disease are full of interest. In 19 children affected by it the temperature was less than 91°·4; in 7 it sank below 78°.8, and the mean of 52 observations is only 87°·8. In extreme cases the temperature may sink to 77°, 74°·3, 72°·5, and in one instance it fell as low as 71°·6. He states that a lowering of temperature precedes the appearance of induration, or at least exists in a very marked degree while the induration is yet very slight. The degree to which the temperature is reduced is always in direct proportion to the degree of the induration, and consequently forms a most important element in the prognosis. In only one case did recovery take place after the temperature had sunk below 90°·5, though life was often prolonged for several days, notwithstanding a much greater reduction of temperature. The slowness of the pulse and respiration likewise bear direct relation to the lowness of temperature and degree of induration, the former having sunk even as low as 60, the latter down to 16 or 14. In connexion with this subject, he likewise alludes to the condition of the lungs met with in this disease, which though called pneumonia, must, as he observes, differ widely from real inflammation of the lungs, since while in true pneumonia the temperature rises even to 105°·8, it sinks in the peculiar condition which attends induration of the cellular tissue as low as 71°·6, and the pulse and respiration become slow instead of accelerated. He regards the state as one of congestion or apoplexy, and expresses the coincidence of his opinion with that of MM. Bailly and Legendre with regard to what they term the "fœtal state" of the lung.

*Icterus neonatorum.* Dr. A. B. Campbell‡ relates three cases of fatal icterus; in two of which the disease depended on congenital absence of the hepatic and cystic ducts; in the third it arose from obstruction of the ductus communis choledochus by inspissated bile. In the first of these cases the icteroid colour of the skin appeared on the day after birth, but the child continued well, though the evacuations were white, until the ninth day. Hemorrhage then took place from the umbilicus, and returned on the following day, when the child died. In this case the gall-bladder was a shut sac, the ducts leading from it being entirely absent, and the blood was tinged with bile. In the second case the symptoms appeared equally early, but no hemorrhage took place at any time. The child wasted while its abdomen enlarged in both hypochondriac regions. Death did not occur till the sixth month; the child having been then attacked with violent diarrhea and vomiting of a fluid, like coffee grounds. The liver was large, the gall-bladder as well as the ducts were absent; the blood and the various tissues were stained with bile. The third case closely resembled the first, hemorrhage from the umbilicus occurred on the seventh day, and returned at intervals till the eleventh, when the child sank into a comatose state and died; the hemorrhage altogether not having exceeded an ounce and a half. In this case all the organs except the liver and spleen were stained with bile, the gall-bladder was full, and the escape of its contents was prevented by a plug of inspissated bile, which occupied the ductus communis choledochus. The brother of this child died at the same age and with similar symptoms.

* Archives Gén. de Médecine, Juin 1845. † Ibid. Mai 1845.

‡ Northern Journal of Medicine, August 1844.

### 4. DISEASES OF SUBSEQUENT CHILDHOOD.

#### DISEASES OF THE BRAIN, NERVOUS SYSTEM, ETC.

The treatise of Dr. Mauthner* on diseases of the brain and spinal cord is one of the most valuable works on this class of diseases that has yet appeared, and deserves to take rank with the treatises of Cheyne and Gölis. It is impossible in this Report to give any abstract of its contents, but a few of the more important observations will be noticed under their proper heads.

A treatise on acute hydrocephalus has been published by Dr. Thomas Smith;† but it contains nothing which need call for further notice.

Dr. Hesse‡ has written an essay on the night-terrors of children, which, though very prolix, is by no means destitute of merit. He has evidently observed such cases with much attention, and describes them very faithfully, but has fallen into the error of endeavouring to make an independent disease out of that which is but a symptom of various morbid states of the system. Hence result an unsuccessful effort at minute diagnosis, and a magnifying of small and accidental differences into important distinctions.

*Acute hydrocephalus.* Dr. Mauthner§ insists much on the differences between this disease and *encephalitis.* Encephalitis is an independent, inflammatory disease which may run its course without any accumulation of serum forming in the ventricles. Hydrocephalus acutus is a secondary affection, resulting from various morbid conditions, of which the tuberculous cachexia is the most frequent. This opinion he supports by the detail of numerous cases, which in this as well as in other parts of the work have evidently been well observed and are well reported. He notices a difference in the order in which the symptoms of the two diseases appear, as affording the ground of distinction between them. Sopor and unconsciousness occur at the onset of inflammation of the brain, but do not appear till the close of hydrocephalus. The former runs its course rapidly and progressively; the latter presents distinct intermissions. Rapid emaciation without assignable cause often precedes hydrocephalus, and it may run its course without the occurrence of convulsions, which convulsions are never absent in inflammation of the brain, and emaciation is not observed till an advanced stage of the disease.

Two cases of fatal encephalitis from insolation are recorded by Mr. Whitehead,|| who appends some judicious remarks on the danger of exposing children with their heads uncovered to the rays of the sun.

In the course of some observations on phthisis, M. Trousseau¶ speaks of those granulations of the membranes of the brain which have been supposed to be due to the presence of tubercle. He states that although the brain is examined in all children who die at the Hôpital Necker, yet these granulations are very rarely met with except in cases where cerebral symptoms have existed during life; but they are always found in greater or less number whenever the patient has died from any head affection. He regards them as fibrinous, not tuberculous, in their nature, and distinguishes the recent granulations from those which are chronic. The former are yellow, soft, and exactly like the masses of fibrine so often met with at the base of the brain, while the chronic deposits are precisely like the old granulations which are often found on the surface of other serous membranes. These conclusions, however, do not quite agree with the results of Dr. H. Lebert's** microscopic researches into the structure of these bodies. He describes them as being composed of the following elements: fibrous tissue derived from the serous membrane around them; tubercle corpuscules situated between the fibres of this tissue; a little granular

* Die Krankheiten des Gehirns und Rückenmarks bei Kinden, 8vo, Wien, 1844.

† On the nature, causes, prevention, and treatment of Acute Hydrocephalus, 8vo; London, 1845.

‡ Ueber das nächtliche Aufschrecken der Kinder im Schlafe, 8vo; Altenburg, 1845.

§ Lib. cit. chapters iii and iv.

|| Medical Gazette, Aug. 23, 1844.

¶ Gaz. des Hôpitaux, Nov. 19, 1844.

** Müller's Archiv, 1844, Heft ii and iii.

matter, and a considerable quantity of a hyaline matter, intermingled with their more solid constituents.

*Hypertrophy of the brain.* Dr. Mauthner's* researches on this subject are by far the most complete that have yet appeared. He prefaces his remarks by a series of observations on the growth of the brain and the increase of its weight at different periods of childhood. He shows that the weight of the organ is greatly modified by the amount of blood which is contained in its vessels, a fact which he uses in support of the opinion that a degree of vascular congestion short of that which would occasion inflammation often gives rise to hypertrophy of the brain. He distinguishes an active and a passive hypertrophy of the brain, the latter of which is attended with expansion of the cranial bones, enlargement of the head, and all that train of symptoms usually regarded as characterizing the affection. In the active form of the disease the bones do not yield, and the first indication of the affection is afforded by the sudden supervention of some form or other of acute cerebral disease. He notices the difficulty of distinguishing between hypertrophy of the brain and *chronic hydrocephalus*, and lays down the following signs as affording means of discriminating between them.† In hypertrophy the posterior part of the skull is that which is observed first to become unnaturally prominent, and the projection of the forehead occurs subsequently, while the projection of the forehead is one of the first results of chronic hydrocephalus. The fontanelles and sutures are never so wide open in hypertrophy of the brain as in chronic hydrocephalus. The latter affection is usually associated with a generally emaciated condition; the former with a leucophlegmatic habit, and with increased deposits of fat. The constitutional symptoms of the two affections likewise differ; convulsions, sopor, and restlessness attend the early stages of chronic hydrocephalus, while spasmodic affections of the respiration are among the earliest indications of hypertrophy of the brain, but seldom occur until an advanced stage of hydrocephalus.

One case of the unsuccessful puncture of the head in a case of chronic hydrocephalus has occurred in the practice of Sir J. Fife.‡ [Of 60 recorded cases in which puncture of the brain has been performed, 17, or 1 in 3½, had a favorable termination, or, in other words, the recoveries have been to the deaths in the proportion of 28 per cent.]

*Convulsions.* Dr. Mauthner's§ chapter on this subject contains many observations of considerable practical value. He points out the fact that convulsions are a frequent result of febrile disturbance in early childhood, and shows how the tendency to venous congestion, which is so characteristic of early childhood, explains this occurrence. Somewhat similar are the remarks of Dr. Morell‖ on the same subject, [though in the form in which he has stated, or rather overstated the facts of the case, he has involved himself in error.] His aim is to show that infantile convulsions are the result of febrile excitement, and that the affection of the brain is in all cases a secondary occurrence. In fever there is increased arterial action on the one side with sluggish venous circulation on the other, connected with imperfect performance of respiration, and a tendency to congestion of the lungs. The imperfect respiration allows the lungs to become congested, and is unfavorable to the return of the blood from the abdomen and head, while the heart by its violent action continues to propel more blood to the brain. The yielding fontanelle relieves the compressed brain for a time, though insufficiently, so that the pressure finally affects the medulla oblongata; respiration is then further impaired, and convulsions result from the spasmodic effort of the voluntary muscles to maintain respiration. Venous blood now pours in rapidly from the extremities to the

* Op. cit. cap. v.

† Ibid. cap. vii, p. 249.

‡ Provincial Medical and Surgical Journal, Nov. 27, Dec. 24, 1844.

§ Op. cit. cap. x.

‖ New York Journal of Medicine, May 1844.

chest; it is prevented, however, from circulating through the lungs by the suspended respiration, the valves of the veins interfere with its return through the channels whence it came; it therefore regurgitates into those veins which are unfurnished with valves, and congestion of the head and abdomen is the result.

Dr. Hennis Green* has recorded the particulars of three cases of what he calls *nervous tremor in children*, an affection unnoticed by any previous writer. It resembled chorea in many respects, but was characterized by a rapid and equable oscillation of the limbs in the direction of flexion and extension, very different from the irregular movements of chorea. One of these cases seemed to have been produced by grief, another by obstruction of the menstrual function, and the third by the poison of lead. They were all unattended by disease of the brain or spinal cord, and were cured by treatment which had reference to the cause that seemed to have produced them. A somewhat similar case is described by Mauthner† under the name of *paralysis agitans*. In this instance, however, it was combined with occasional tetanic seizures, and seemed to depend on an inflammatory condition of the spinal cord.

Dr. Lichtinger,‡ in a series of papers on stuttering, distinguishes those cases which depend on affection of the nervous system from such as result from disease or malformation of the organs of speech or respiration. He distinguishes further *cerebral and spinal stuttering*. In the former, disease of the brain interferes with the efforts of the will, and the activity of the spinal cord preponderates unchecked, unregulated. Spinal stuttering must be referred to morbid action of that portion of the spinal cord situated between the origin of the fifth and seventh nerves, and those respiratory nerves that supply the muscles of the chest and belly. This may be either central, in which the cause exists within the above-named tract of the cord; or eccentric, in which the cause is seated in some of the reflex nerves, or much more rarely in the motor nerves. He relates two interesting cases of central spinal stuttering, both of which succeeded to injury of the upper part of the spine, and were attended with convulsive movement of some of the limbs. One of these cases terminated fatally, and a post-mortem examination disclosed softening of the upper part of the spinal cord. He notices the frequency of reflex stuttering, in which the affection depends on irritation in some distant organ, and has collected 76 cases of this variety, in three fourths of which the source of irritation was seated in the abdomen.

### DISEASES OF THE ORGANS OF RESPIRATION AND CIRCULATION, AND OF THEIR APPENDAGES.

*Pneumonia.* One of the most important contributions that has been made of late to our knowledge of infantile disease is the essay§ of MM. Bailly and Legendre on what is usually termed *lobular pneumonia*. They attack the generally received opinion of the inflammatory nature of this condition, which they regard as analogous to the state described by Jörg as atelectasis pulmonum. They conceive that this which they call the "*fœtal state*" of the lung is not invariably congenital, but that it may supervene afterwards under certain circumstances. The following conclusions embody the more important results of their researches. 1. In the bodies of children who have been rhachitic, weakly, or exhausted by previous disease, a number of lobules of the lungs are found in a peculiar state of condensation, similar to that of the fœtal lung. 2. This fœtal state, which consists in occlusion of the vesicles, may result from the mere contractility of the tissue, or may depend on congestion of the vascular network exterior to the vesicles. The former is the simple, the latter the congestive form of this affection. The congestive form is usually met with

* Provincial Medical and Surgical Journal, Feb. 24, 1844.

† Op. cit. s. 441.

‡ Med. Zeitung, Aug. 14, 21, 28, 1844.

§ Arch. Gén. de Méd. Janv., Févr., Mars 1844.

along the posterior border of the lungs, and generally accompanies catarrhal inflammation of the pulmonary vesicles. 3. In either of these forms of the fœtal state, insufflation reproduces more or less completely the natural condition of the lobules. 4. Though occasionally met with unassociated with inflammation, yet in by far the majority of cases this condition becomes developed under the influence of catarrh and catarrhal pneumonia. 5. When unattended with catarrh and involving only isolated lobules, this condition cannot be detected till after death, but in the new-born infant it usually affects the lobar form, is attended by the physical signs of deficient respiration, and associated with the absence of all signs of constitutional reaction. 6. It is essentially different from hepatization, is produced by causes which interfere with the free performance of respiration, and is to be treated by remedies the reverse of antiphlogistic. 7. Lobular pneumonia has, strictly speaking, no existence, since the action of inflammation is never confined to a single lobule, as is the case with the fœtal state of the lung. *Partial pneumonia* would therefore be a fitter term. 8. Insufflation does not modify the patches of true hepatization, while the bronchi leading to such hepatized nodules are exempt from catarrh; two characters which distinguish partial pneumonia from the lobular engorgements of catarrhal pneumonia. 9. True partial pneumonia is by no means common in children, though when hepatization does occur in children under 5 years of age, it almost always affects the partial form. The statements, therefore, that have been made with reference to the rarity of lobar pneumonia in infancy are correct; but almost all that has been said about the extreme frequency of lobular pneumonia at that age must be taken as referring to the fœtal state of the lung. 10. Catarrhal pneumonia consists in the extension of the catarrhal inflammation from the bronchi to the pulmonary vesicles. This inflammation may affect healthy lobules, or those in the fœtal state. In the latter case it gives rise to appearances which have led to the supposition that these lobules were the seat of a parenchymatous inflammation. 11. Capillary bronchitis and generalized lobular pneumonia are but two forms of catarrhal pneumonia, which differ according as in the one the catarrhal element or as in the other the lobular congestion predominates. 12. These facts explain why depletion was seldom appropriate in the treatment of what was called lobular pneumonia. [Simple as the process was by which these results were obtained, no one had previously employed insufflation as a means of ascertaining the real nature of lobular pneumonia and carnification of the lung in children. The writer of this Report has repeated the experiments of MM. Bailly and Legendre on many occasions, and can fully substantiate the correctness of their statements. An assertion has been made by M. Bouchut, that even true hepatization may be removed by insufflation;* in this, however, he is decidedly wrong. The hepatized portion may sometimes be made to assume a brighter colour, but not to resume the texture of healthy lung, as is the case with lung in the fœtal state.] Dr. Posner,† in some remarks on the treatment of pneumonia in childhood, observes that the strictly antiphlogistic treatment suitable to the inflammatory affections of the adult, are no longer appropriate in early life. He applies these observations especially to pneumonia, in the course of which an adynamic stage comes on, requiring the discontinuance of other remedies, and the use of wine and stimulants, for the employment of which he lays down clear and sensible directions.

*Hooping-cough.* Dr. Lersch‡ confirms the statements of some previous writers with reference to the existence of small ulcerations about the root of the tongue in hooping-cough. He does not know whether their formation is preceded by the appearance of vesicles in that situation, for he has always seen them having the character of small ulcerations from one to three lines broad,

* Op. cit. p. 317.

† Journal für Kinderkr. März 1844.

‡ Allg. Med. central Zeitung, Sept. 18, 1844.

slightly excavated, of a circular form, and situated at the insertion of the frænum. He appears to wish to establish an analogy between hooping-cough and hydrophobia, on the somewhat slender ground of the paroxysmal character of the two diseases, and the presence of vesicles under the tongue in hydrophobia, which may be analogous to the ulcerations in that situation in hooping-cough. M. Levrat Perroton* recommends the liquor ammoniæ in hooping-cough; but gives no stronger evidence of its utility than is afforded by four imperfectly reported cases, in all of which depletion had previously been practised. Dr. Panck† details the results of trials of various remedies in hooping-cough. In some cases he found hydrochloric acid very useful after the subsidence of the inflammatory stage, especially when the cough was attended with frequent vomiting of diseased mucus from the stomach. He employs the dilute acid in doses of about ten minims every hour. In the same stage of hooping-cough Dr. Golding Bird‡ has employed alum, in doses of two or three grains every four or six hours for a child of 3 years old, and believes that it exerts a specific action on the disease. Dr. Dieudonné§ writes in praise of cochineal in hooping-cough, [an old English remedy, to which Dr. Cajetan Wachtl called attention on the Continent some three years ago; but which, during a very patient trial of its merits at the Infirmary for Children, the writer of this Report found to be almost inert.]

*Retro-pharyngeal abscess.* A well-marked instance of this affection has been related by Dr. O'Ferrall.‖ It occurred in an infant aged 4 months, who was saved from impending suffocation by puncture of the swelling, a proceeding which it was necessary to repeat several times. He recommends the use of a bistoury, with a short cutting edge, as a preferable instrument to a trocar, for opening these tumours, since their tough parietes do not very readily yield, and the trocar may strike upon the vertebral column before penetrating their walls.

*Phthisis.* Dr. Hennis Green¶ has drawn up a tabular view of the seat of tubercle in 180 cases of tubercle of the lungs in children. The table is preceded by some remarks on pulmonary phthisis in the young subject, which are confirmatory of the statements of MM. Rilliet and Barthez, but do not contain anything new.

### DISEASES OF THE ABDOMINAL VISCERA.

*Atrophia ablactatorum.* Dr. S. S. Alison** describes a peculiar state of the stomach, which he met with in a child who died at the 3d month, having been weaned when a month old, and subsequently fed with unsuitable food. The child had an insatiable hunger, vomited much, and suffered from abundant feculent diarrhea. The stomach was only two inches long, and weighed only ℨiss. Its walls were thickened, but otherwise healthy, and the duodenum was similarly contracted. He attributes this condition to muscular action, excited by the irritating food which the stomach in a measure rejected, while the rest of the food remained too short a time to undergo changes into chyme or chyle. Dr. Weisse†† recommends in those cases of diarrhea with rapid emaciation, which come on after weaning, that if it be not possible to procure for the children a good nurse, they should be supplied with raw beef finely shred, of which they should take two tablespoonsful divided into four parts in the course of 24 hours; the quantity being afterwards gradually increased to as much as they will take. He states that gradual cessation of the diarrhea, and recovery of flesh, are the results of the treatment. He proposes to experiment on the use of pure osmazome, since he has found in these cases the animal fibre, nearly unchanged in the evacuations.

* Revue Médicale, Juin 1844.
† Oppenheim's Zeitschrift. Feb. 1845.
‡ Guy's Hospital Reports, April 1845.
§ Journal für Kinderkr. Juli 1844.
‖ Dublin Hospital Gazette, March 1, 1845.
¶ Medico-Chirurgical Transactions, vol. xxvii, p. 351.
** Medical Gazette, Nov. 1, 1844.
†† J. f. Kinderkr. Feb. 1845.

*Diarrhea.* M. Trousseau* has made some remarks in one of his clinical lectures on *cholera infantum*, and these observations are reproduced more fully in the work of M. Bouchut.† The anatomical characters of the affection are very minutely described by him. In its treatment he attaches considerable value to the nitrate of silver, and an equally favorable account of its utility in diarrhea both acute and chronic, is given by Dr. Henock,‡ who watched its employment in Professor Romberg's clinic at Berlin.

*Incontinence of urine.* Dr. Delcour§ in the course of some remarks on the nocturnal incontinence of urine in children, recommends benzoic acid and nitrate of potash as two very valuable remedies for the affection. Dr. Delcour employs the nitrate of potash, which was first introduced into practice in these cases by Dr. Young of Chester, in doses of ʒss daily, for children of 7 years of age. He was induced to try the benzoic acid by its known action on mucous membranes, and relates two cases in which recovery took place during its employment, after both nitre and strychnine had failed.

### FEVERS.

*Measles.* Dr. Seidl‖ describes an epidemic of measles which prevailed in the year 1840, in the district of Zolkiew in Austria. It was extensive and fatal, having attacked 1519 persons out of a population of 32,736, and having proved fatal to 196, or 12·8 per cent. of those who were attacked. Children, especially from the age to 4 to 12, were most frequently affected by it; no one above 40 years old suffered; but many young women were seized by it, either just before or immediately after delivery. The disease made its appearance in the autumn of 1839, but disappeared during the early part of the winter, breaking out again in January 1840, reaching its acme in April and May, and ceasing in September. The milder forms of the disease presented no peculiarity; the severer cases began very tempestuously, and were attended with affection of the pharynx and larynx, or of the brain and its membranes. It was often complicated with diarrhea, which in most cases was followed by no ill consequences, though occasionally it became chronic, and then led to bad results. It was, however, a more fatal complication than that with hooping-cough, which in some instances ran its course nearly simultaneously, and ceased at exactly the same time with the termination of the period of desquamation. Cancrum oris occurred as a sequela of the disease five times, diarrhea frequently, and general dropsy often came on during the stage of desquamation just as it does in scarlatina. When this state of anasarca followed diarrhea, the prognosis was usually unfavorable; but diarrhea appearing subsequently, was in general salutary, and often removed the dropsy. Dr. Cathcart Lees¶ describes some of the more dangerous complications of measles as observed by him during an epidemic which prevailed among the children in the South Dublin Union Workhouse. The children had been much crowded together, and were not in a good state of health when the disease broke out. Of 48 children under 2 years of age, 18 died; of 35 between 2 and 5, 6 died; and of 112 between the ages of 5 and 12, 5 died. Pneumonia was a very frequent complication, especially among the younger children, having been present in 40 out of 48 infants under 2 years of age. The epidemic was likewise attended with a fatal affection of the throat, [which appears to have been very similar to that described by the writer of this Report in the Medical Gazette, for August 25, 1843,] that occasionally appeared as early as the second day of the eruption, though usually at the period of its decline. Some of the cases which presented peculiarities in their course, are related in full; among which are two instances of sloughing of the labia and rectum, similar to those re-

* Gaz. des Hôp. Fev. 1, 1844. † Op. cit. p. 210. ‡ Journal f. Kinderkr. Juli 1844.
§ Gaz. des Hôpitaux, Dec. 21, 1844. ‖ Oesterr. Med. Jahrb. Dec. 1843, p. 263.
¶ Dublin Journal, Sept. 1844.

corded bv the late Mr. Kinder Wood. A dissertation has been published by Dr. Geerstema,* the aim of which is to prove that measles and scarlatina are only modifications of the same morbid poison. In support of this opinion, he appeals to the statements of previous writers, as also to the evidence afforded by some cases which came under his own observation during an epidemic of measles at Groningen, in 1842. His cases, however, appear to be instances of the supervention of the one disease on the other, rather than to afford evidence of any closer affinity between the two.

*Scarlatina.* Mr. Porter† describes a singular succession of rheumatic symptoms attended by protrusion of the right eye, which succeeded to a mild attack of scarlatina, all of which ceased suddenly on the supervention of pericarditis. Blisters, calomel, opium and colchicum, were succeeded by restoration to health, but the vision of the right eye was lost, and the organ became atrophied. Dr. S. S. Alison‡ has treated of the occurrence of pericarditis in scarlatina, a complication which he thinks has not sufficiently engaged the attention of medical observers. He relates three cases in which he assumes this affection to have existed. The first case was not seen by him until a week before death; three months had then elapsed since the occurrence of scarlatina. The right side of the chest was full of sero-purulent fluid, and ℥ vj of a similar fluid were contained in the pericardium, between which and the heart there existed a few adhesions. Neither the second nor third case can be regarded as conclusive, since in neither was there heard any morbid sound; but increased impulse and violent palpitation of the heart are the only grounds on which he supposes it to have existed. Mr. Snow§ calls Dr. Alison's attention to the account which he published of three cases of pericarditis after scarlatina, in the Lancet for December 14, 1839. He regards Dr. Alison's assumption of the early occurrence of pericarditis in connexion with scarlatina as unfounded. He conceives the pericarditis to be the result not of the fever, but of the renal disease which succeeds to it; and which may give rise to pericarditis wholly independent of the previous occurrence of scarlatina.

*Variola, varioloid diseases, and vaccination.* Dr. L. Wagner‖ describes a very mild *epidemic of smallpox* in the district of Neufeld, near the Danube. The disease attacked 2509 out of a population of 70,000, who resided in a district containing 20 square miles. Of those who suffered from the disease, 102 only had been vaccinated, and but 9 of these presented well-marked cicatrices. The disease was very mild even in those who had not been vaccinated since the total mortality did not exceed 222 or 8·7 per cent. In all the vaccinated, the disease ran a modified course, and only 1 of them died. In many cases when vaccination was practised in consequence of smallpox having occurred in the house, variola appeared on the 2d or 3d day afterwards, while at the same time the vaccine vesicle ran a perfectly normal course. Dr. Woppisch¶ gives the particulars of an epidemic of smallpox at Zeitz, in 1841, which appear to him to support the opinion of the *identity of varioloid and variola.* The facts on which he founds his opinion, are that the first two cases which occurred, were cases of varioloid in two vaccinated children, the next in the same house was a case of variola in an unvaccinated child. At the commencement of the epidemic, the vaccinated suffered exclusively from varioloid, the unvaccinated from variola, but as the disease grew more prevalent, varioloid occurred likewise among the unvaccinated. Most of the cases of variola occurred in unvaccinated children under the age of 1 year, not a single vaccinated child under 7 years had true variola, and only 12 a very mild form of varioloid. Up to the

* De affinitate morbillorum cum scarlatina; Groningæ, 8vo. The writer of this Report has been unable to obtain this essay; and therefore judges of it from an abstract in the Journal f. Kinderkr. Aug. 1844.

† American Journal of Medical Science, Jan. 1845.

‡ Medical Gazette, Feb. 21, 1845.

§ Ibid. March 7, 1845.

‖ Oesterr. Med. Jahrb. Nov. 1844.

¶ Med. Zeitung, Feb. 28 and March 20, 1845.

age of 14 indeed, all the vaccinated children who were attacked, had a very mild varioloid; while persons between the ages of 20 and 40, although vaccinated in their infancy, had confluent varioloid closely resembling smallpox. From these facts, Dr. Wagner infers that the varioloid is smallpox mitigated by vaccination. This conclusion, however, is opposed to observations made apparently with equal care by Dr. Fischer* of Tambach, in the Duchy of Gotha, who observed an epidemic of *varioloid quite independent of smallpox*, but alternating with epidemic scarlatina. He founds his opinion as to the non-affinity of the two diseases, on 1st, the shorter duration of the eruption, the fact that it appeared first on the extremities, and that it was always succeeded by desquamation of the skin. 2d. The absence in its course of any affection of the conjunctiva. 3d. The invariable occurrence of erythema before the eruption, and the fact that the red spots of the early eruption, had not the central hardness of variola. 4th. The absence of smallpox odour, or of the suppurative fever, and the desiccation of the pustules on the 6th, not on the 8th or 9th day after their appearance. 5th. The circumstance that the course of the attack was in no degree modified by previous vaccination. 6th. The very mild character of the epidemic. M. Legendre† has investigated the very difficult subject of the *simultaneous existence of variola and vaccinia*, of which he has observed 10 instances. His conclusions, which are founded on a comparison of 56 observations derived from different sources, are to the effect that vaccination almost always modifies the characters of variola, but that the performance of vaccination in a child previously exposed to the contagion of smallpox, seems to favour the appearance of that disease, though in children above 4 years of age it usually appears in a favorable and greatly modified form. That while vaccination performed during the incubation of smallpox, modifies the characters of that disease, the vaccine vesicle itself is usually modified in a degree directly proportionate to the shortness of the interval between the performance of vaccination, and the appearance of smallpox. When vaccination is performed after the appearance of variola, the vaccine vesicle sometimes runs its course, but does not modify the variola. The practical inference which he deduces, is that in young and weakly children who have been exposed to the contagion of variola, the performance of vaccination only increases their danger, and is therefore to be avoided. Mr. Wylde‡ brings evidence of the *good results of vaccination in Ireland*, from the tables drawn up during the census of that kingdom in 1841. He shows that, notwithstanding the vast increase of the population of Dublin, the deaths from smallpox during the past 10 years have scarcely amounted to half of the number who died from the same cause in an equal space of time during the middle of the last century. He states further that the superiority of vaccination over inoculation is shown by the fact that smallpox mortality is highest in those provinces in which inoculation is most practised, and vaccination least. The proportion borne by smallpox to all other epidemic diseases is—

| | | | | | |
|---|---|---|---|---|---|
| Leinster .. | 1:8·9 | Ulster .. | 1:5·96 | Dublin .. | 1:13·79 |
| Munster .. | 1:6·6 | Connaught .. | 1:5·35 | | |

[This, however, is not of itself proof of the rarity of smallpox in Leinster or Dublin. It may result, and in Dublin it doubtless does, in part from the greater frequency of typhus and other epidemic diseases.]

The comparatively small *success of vaccination* in India, has given rise to an inquiry, the results of which are contained in the valuable Report of Dr. Duncan Stewart.§ The chief causes of this want of success may be referred to the heads of—1st. Native prejudice. 2d. The propagation of a spurious disease owing to the carelessness of native vaccinators. 3d. The influence of

* Casper's Wochenschr. Dec. 28, 1844.

† Arch. Gén. de Méd. Sept. 1844.

‡ Edinb. Med. and Surg. Journal, April 1845.

§ Report on Smallpox in Calcutta and Vaccination in Bengal, 8vo; Calcutta 1844. A fuller notice of this interesting document will be found in the present Number of this Journal.

climate, which for about six months in the year renders the vaccine vesicle imperfect, and for three out of those six months so modifies the virus as usually to render vaccination altogether unsuccessful. 4th. The fact that this influence of climate varies much in different parts of India, coming into operation in some places as early as March, in others not till two or three months later; and the additional fact that a similar variation will take place at the same locality without any known cause. 5th. The existence of some constitutional peculiarity in the natives, which renders them indisposed to the reception of the vaccine virus, or at least interferes with the full development of the vesicle; and renders the protection afforded by vaccination imperfect, as is shown by the fact that smallpox after vaccination occurs in a grave form more frequently in natives of India than in Europeans. In a paper read before the Medico-Chirurgical Society,* Dr. Gregory mentions some facts which transpired during the smallpox epidemic of 1844, and which show the preservative powers of vaccination in a rather questionable light. He states that the deaths from smallpox in London were during its prevalence as numerous as 60 years ago, that half of the patients who were received into the Smallpox Hospital had distinct cicatrices of vaccination, and that 8 per cent. of them died. Since then at least 7 per cent. of those who have smallpox after vaccination die, while the deaths from inoculated smallpox do not exceed 1 in 500; he is disposed to recommend inoculation as a test of a person's safety more satisfactory than revaccination. He would therefore wish for such a modification of the present government regulations as would allow of the performance of variolous inoculation of persons between the ages of 10 and 20, as a test of the success of their previous vaccination, and of the persistence of its protective power. M. Calosi† makes the assertion, unsubstantiated, however, by confirmatory documents, that of 38,137 inhabitants of Tuscany, vaccinated in infancy whose ages varied from infancy to 34 years, many have been revaccinated without success, and none have contracted variola, though among them are several who have frequently been exposed to its contagion. He hence draws a conclusion adverse to the supposition that the protective power of vaccination becomes impaired by time, and consequently adverse to the practice of revaccination. The question of the degeneracy of the vaccine virus, of the decline of its protective power by the lapse of time and of the *utility of revaccination,* continues to engage much attention on the continent. Revaccination is still practised annually throughout the whole Prussian army, with a tolerably uniform result, the disease being produced in about 50 per cent. of those who are vaccinated.‡ M. Villaret§ has published an account of a series of revaccinations carried on during four years in a regiment of dragoons. The following are the results which he obtained:

| | Number vaccinated. | With success. | Unsuccessfully. |
|---|---|---|---|
| Had had smallpox | 273 | 183 | 90 |
| Had been previously vaccinated { with success | 848 | 716 | 132 |
| Had been previously vaccinated { without certain success | 124 | 94 | 30 |
| Had not had smallpox, nor been vaccinated | 160 | 150 | 10 |
| | 1405 | 1143 | 262 |

Dr. Condie‖ has from various sources compiled tables which yield 133,042 successful revaccinations, and 53,654 spurious vesicles out of 346,583 revaccinations. It further appears that out of 220,818 persons who were revaccinated, 173,659 had perfect cicatrices, 32,418 imperfect, and the remainder had none at all. In 87,399 of these, revaccination was perfectly successful; and on subjecting 61,746 of those in whom it had failed to a second revaccination perfect vaccine vesicles were produced in 9,238. Almost all writers on

* On Jan. 28, 1845; reported in the Medical Gazette, Feb. 7, 1845.

† Bulletino dell Scienze Mediche, Giugno 1844.

‡ The results of the year 1843 are contained in Med. Zeitung, April 3, 1844; those of 1844, Ibid., April 9, 1845.

§ Gazette Médicale, Mars 2, 1844.

‖ Op. cit.

the subject agree in advocating revaccination, and likewise coincide pretty nearly in the arguments they adduce in its favour. The late Dr. Forry,* whose premature death is a loss to medical science, M. Schäffer,† Dr. Losetti,‡ and the candidates for the prize offered by the French Institute for the best essay 'on vaccination and its influence on smallpox,'§ agree on this point. Their main arguments are derived from the fact that while smallpox occurs but seldom after vaccination in children under ten or twelve years, its attacks are much more frequent after this period, and increase both in frequency and severity up to about the age of 35, when the constitution seems to acquire a comparative insusceptibility to the poison of variola. Hence they deduce the practical inference that a second vaccination should be performed at about the age of 15; and its repetition again at 25 has not been without its advocates. Of course there are many facts by which they support their opinion, as well as some objections that might be raised to their inferences, mention of which is prevented by the limits of this Report.

Dr. Fiard|| inquires into the alleged *degeneracy of vaccine virus* by its transmission through many individuals. He is a believer in the reality of this occurrence; the first indication of which he thinks is presented by the diminution in the duration of the eruption as compared with that of an earlier date, and that a difference in the development of the vesicle on the 8th or 9th day is not observed till afterwards. He applies this hypothesis to the vaccine virus of 1836; the vesicle from which runs the same course with that produced by the virus of 1844 up to the 8th day. At the 9th day desiccation of the vesicles of the old vaccine commences, and is complete by the 13th or 14th day, while the new runs its course more slowly and its desiccation is not complete till the 16th or 17th day. He states that a similar difference was observed some years ago between the vesicles resulting from the old Jennerian vaccine, and the then new virus of 1836. Dr. v. Fradeneck¶ has *discovered the original vaccinia* among some cows in part of Carinthia. The vesicles which it produced differed in no respect from those which resulted from the old virus, a fact from which he draws inferences unfavorable to the alleged degeneracy of the vaccine matter.

Dr. Pluskal** gives an account of a series of experiments on *retrovaccination*, which he carried on for several years on a great variety of animals. It appears that it was only in those animals in whom vaccinia occurs spontaneously that vaccination was followed by the appearance of characteristic vesicles, and that the experiment succeeded best in those animals which were most nearly allied to the ox tribe. He regards the results of retrovaccination when practiced carefully on the cow, as affording a good criterion of the goodness of the lymph. but does not believe in its utility as a means of regenerating a deteriorated virus.

Dr. A. F. Tassani†† relates a very singular history of the apparent *communication of syphilis* to several infants by *vaccinating* them from a child in whom syphilitic symptoms subsequently appeared, though no sign of any such disease existed at the time when vaccination was performed.

Dr. Osbrey‡‡ relates two cases of *gangrenous inflammation of the vaccine vesicle* coming on about the 12th day, and proving perilous, though not fatal to two children, one of whom was 18 months, the other 5 years old. In one of the cases in addition to the local gangrene, sloughing of the mucous membrane of the mouth occurred, attended with hemorrhage from it. Recovery was slow in both instances. No cause could be assigned for the occurrence, as the previous health of both children had been good. [Dr. Osbrey quotes Dr. Labatt as mentioning this accident, but is apparently unacquainted with

* New York Journ. of Med. Sept. 1844.

† Med. Zeitung, March 27, 1844.

‡ Gaz. Méd. de Paris, 11 Mai 1844

§ Revue Médicale, Mars 1845.

|| Bull. de l'Acad. Roy. de Méd. Nov. 30, 1844.

¶ Oesterr. Med. Jahrb. Mai 1844.

** Oesterr. Med. Wochenschr. März 1844.

†† Gaz. Med. di Milano, Ottobre 14, 1843.

‡‡ Dublin Medical Journal, March 1844, p. 133.

other instances of the occurrence of erysipelas after vaccination. F. de Wuerst, in his dissertation De erysipelate Neonatorum post Vaccinationem, Dorpat, 1835, 8vo, mentions twenty cases, and gives references to several recorded by different writers. In none of Wuerst's cases, however, did gangrene occur; the children dying as from ordinary erysipelas.]

DYSCRASIÆ, ETC.

*Scrofula.* The first part of a treatise on scrofula has appeared from the pen of M. Lugol.* The influence of hereditary predisposition in giving rise to the disease is treated of most fully, and amply illustrated from M. Lugol's large experience. It may be doubted, however, whether due weight is attached to the other causes of this disease. M. Négrier† has published a second account of his experiments on the treatment of scrofula by walnut tree leaves. He states that of the 55 persons treated by this remedy, whose cases he mentioned in his first Report, 34 continue radically cured, and he considers the results of his second series of experiments as fully confirmatory of his former opinion. The action of the remedy is very slow, its use for from 20 to 50 days being requisite before any effect becomes apparent, and its employment seems to have been continued in some instances for 6 or 8 months. He states, however, that relapses are very rare. It acts most slowly in scrofulous swellings of the glands, but very rapidly on diseased bones, or ulcerated surfaces, or in strumous ophthalmia, in which a decoction of the leaves employed as a collyrium is extremely serviceable.

*Rickets.* M. Trousseau‡ strongly advocates the use of the oleum jecoris aselli, which he gives in doses of ℈j to ʒiij daily, mixed with sugar or syrup. He looks for some obvious improvement in 8 or 10 days, and for cure in the course of a month or six weeks. He insists much on the observance of a milk diet, and on abstinence from meat during the treatment, and disapproves of all orthopedic proceedings for straightening the limbs.

*Cretinism.* Much interest has been excited by Dr. Guggenbühl's philanthropic efforts to cure this distressing malady. His first Report§ has been published, in which he makes some observations on the affection, and describes his mode of treatment. The period of liability to cretinism extends from dentition to the 7th year. Its curability is greatest during the first two years of its existence, and appears afterwards to be in direct relation to the power of speech. Idiocy and cretinism are by no means synonymous terms, and cretinism does not depend simply on want of cerebral development. A morbid condition of the system generally, allied to struma or rickets is the foundation of the disease, to which the decay of the intellect is superadded. Defective cerebral development is often associated with cretinism, and one remarkable case is related in which the head of a child aged two years and a half, which had measured 15 inches round at the time of its reception into the institution, gained $2\frac{1}{2}$ inches in circumference during a sojourn of 30 months upon the Abendberg. The hospital is situated at a height of 3000 feet, in the midst of the Bernese Alps, between the lake of Thun and Brienne. Most of the patients are children under 1 year old. Dr. Guggenbühl's first aim is to improve their physical condition. For this purpose he uses daily bathing in water, impregnated by means of an electro-magnetic apparatus. The oleum jecoris aselli, iodide of iron, and quinine are the chief medicines employed. Much importance is attached to keeping the children in the open air. Goat's milk is much used as an article of diet. Great caution is observed in proceeding to the mental education of the patients; and the education of the senses is that which is first attempted. A pictorial grammar by Czech is much used in teaching the elements of speech.

* Recherches sur les causes des Maladies Scrofuleuses; Paris, 1844, 8vo. An English translation has been published by Dr. Ranking.

† Archives Gén. de Méd. Fév. 1844.

‡ Journal de Médecine, and Journal f. Kinderkr. März 1845.

§ L'Abendberg, établissement pour la guérison et l'éducation des enfans crétins, 8vo; Fribourg, 1844.

